# The CRISPR/Cas-associated scaRNA modulates *efeUOB* expression and stress responses in *Neisseria meningitidis*

**DOI:** 10.1093/femsml/uqag027

**Published:** 2026-07-20

**Authors:** Denise Pytlik, Milan Gerovac, Thorsten Bischler, Andreas Schlosser, Jörg Vogel, Christoph Schoen

**Affiliations:** Institute for Hygiene and Microbiology (IHM), University of Würzburg, 97080, Würzburg, Germany; Helmholtz Centre for Infection Research (HZI), 38124, Braunschweig, Germany; Core Unit Systems Medicine, University of Würzburg, 97080, Würzburg, Germany; Rudolf Virchow Center for Integrative and Translational Bioimaging (RVZ), University of Würzburg, 97080, Würzburg, Germany; Institute for Molecular Infection Biology (IMIB), University of Würzburg, 97080, Würzburg, Germany; Helmholtz Institute for RNA-based Infection Research (HIRI), 97080, Würzburg, Germany; Institute for Hygiene and Microbiology (IHM), University of Würzburg, 97080, Würzburg, Germany

**Keywords:** CRISPR/Cas, scaRNA, *Neisseria meningitidis*, virulence, oxidative stress, iron

## Abstract

*Neisseria meningitidis* is a human-adapted commensal pathogen that must continuously balance nutrient acquisition with stress tolerance. Here, we identify a type II-C CRISPR/Cas-associated small RNA (scaRNA) as a posttranscriptional regulator of the *efeUOB* operon and oxidative stress responses. Using *in vitro* RNA binding and structure probing assays, we show that the scaRNA interacts with the 5′ untranslated region of *efeO* mRNA, leading to reduced translation of this component of the ferrous iron transporter EfeUOB. Consistent with this, *efeO* translational fusions demonstrate repression by the scaRNA, whereas a ΔscaRNA mutant shows increased reporter expression. We further show that meningococcal Cas9 (Nme1Cas9) is able to cleave scaRNA *in vitro*, but *in vivo* phenotypes are primarily scaRNA-dependent, indicating that Nme1Cas9 contributes, at most, indirectly to this regulation. In line with this observation, comparative proteomics revealed overlapping but distinct roles of scaRNA and Nme1Cas9 in oxidative stress adaptation, energy metabolism, and ion transport. While steady-state protein abundances did not capture all scaRNA-dependent effects, functional assays confirmed that scaRNA inactivation reduces survival under oxidative stress. Together, our results identify scaRNA-mediated repression of *efeO* as a novel posttranscriptional mechanism that contributes to stress adaptation in meningococci. These findings expand the functional repertoire of CRISPR-associated elements and suggest a role for small RNA-based regulation in iron-related stress adaptation in a major human pathogen.

## Teaser

The small scaRNA molecule, together with the Cas9 protein, helps *Neisseria meningitidis* adjust how it takes up iron and copes with stress from harmful oxygen compounds.

## Importance

*Neisseria meningitidis* is a leading cause of bacterial meningitis and must adapt to fluctuating nutrient availability and host-imposed stress conditions. Here, we identify a CRISPR/Cas-associated small RNA (scaRNA) that represses translation of the iron transporter component *efeO* and thereby modulates oxidative stress tolerance. Functional assays demonstrate that scaRNA inactivation reduces survival under oxidative stress, and proteomics reveal partially overlapping but distinct contributions of scaRNA and meningococcal Cas9 (Nme1Cas9) to metabolism and ion transport. While Nme1Cas9 is able to cleave the scaRNA *in vitro, in vivo* phenotypes are driven primarily by scaRNA. These findings highlight a role for CRISPR-associated small RNAs in posttranscriptional regulation of stress adaptation, with physiological implications for iron metabolism.

## Introduction

The Gram-negative bacterium *Neisseria meningitidis* (Nme) has long been recognized as a highly successful organism that exclusively colonizes humans (Rouphael and Stephens 2012). The evolution of *N. meningitidis* as a human-specific commensal and an occasional fulminant pathogen represents an important case study in microbial pathogenesis (Caugant and Maiden 2009, Mullally et al. 2021). Despite the availability of vaccines, *N. meningitidis* remains a major human pathogen and a leading cause of meningitis worldwide (GMAR 2023, Kwambana-Adams 2023). The persistence of meningococcal disease is largely due to the organism’s significant genetic plasticity and antigenic variability, making *N. meningitidis* one of the most genetically diverse bacteria known (Caugant and Maiden 2009, Caugant and Brynildsrud 2020). This diversity arises from multiple mechanisms of horizontal gene transfer and mutation (Rotman and Seifert 2014). The resulting variability in surface-exposed structures facilitates immune evasion and functional variation, with hypervirulent lineages often associated with elements such as the meningococcal disease-associated prophage (MDAΦ) (Bille et al. 2005, 2017, Meyer et al. 2016) and the capsule polysaccharide synthesis cluster (Bille et al. 2005, Brynildsrud et al. 2018, Mullally et al. 2021).

The horizontal transfer of genetic material in *N. meningitidis* is, amongst others, limited by a type II-C clustered, regularly interspaced, short palindromic repeat (CRISPR)/CRISPR-associated protein (Cas) system (Zhang et al. 2013) (Fig. S1). CRISPR/Cas loci, which function as adaptive immune systems in prokaryotes, rely on RNA-guided nucleases to limit the acquisition of foreign genetic material (Hille et al. 2018). In type II systems, CRISPR loci are transcribed and processed into CRISPR RNAs (crRNAs) that base-pair with trans-activating CRISPR RNAs (tracrRNAs). These RNA duplexes form a complex with the endonuclease Cas9 to bind and cleave double-stranded DNA complementary to the crRNA, flanked by a protospacer-adjacent motif (PAM) (Leenay and Beisel 2017). Type II CRISPR/Cas systems are of particular medical interest as they are associated with human pathogens such as *Campylobacter jejuni* (Cje) (Lowen et al. 2013, Saha et al. 2020), *Francisella novicida* (Fno) (Joseph et al. 2011, Sampson et al. 2013, 2019), and *N. meningitidis* (Roberts 2008, Rouphael and Stephens 2012), where they have been shown to play roles in virulence and gene regulation (Ratner et al. 2015).

The meningococcal type II-C system, present in ∼60% of meningococcal strains, is the most streamlined CRISPR/Cas system characterized to date and is located on a mobile genetic element (Zhang et al. 2013). Three variants of meningococcal Cas9 (Nme1Cas9 to Nme3Cas9) exist in a strain-specific manner (Edraki et al. 2019). Nme1Cas9 has been shown to function also as a single-guide RNA (sgRNA)-driven genome-editing tool in human cells (Esvelt et al. 2013). In addition to its dual and single RNA-guided DNA nuclease activity, Nme1Cas9 cleaves ssDNA in a tracrRNA- and PAM-independent manner (Zhang et al. 2015), and *in vitro* possesses an RNA-guided, PAM-independent ssRNase activity (Rousseau et al. 2018).

Beyond its ‘canonical’ role in defending against foreign DNA, the so-called ‘noncanonical functions’ of Cas9 are increasingly recognized for their role in regulating bacterial virulence. In *C. jejuni*, CjeCas9 binds and cleaves endogenous mRNAs in a tracrRNA- and crRNA-dependent but PAM-independent manner, regulating ∼100 transcripts, including flagellin glycosylation genes (Sampson et al. 2013, Dugar et al. 2018). Similarly, in *F. novicida*, FnoCas9, together with a small CRISPR/Cas-associated RNA (scaRNA), represses a specific regulon of four genes that must be silenced for virulence. This regulation occurs through a tracrRNA- and PAM-dependent interaction with endogenous DNA (Ratner et al. 2019). In *N. meningitidis*, Nme1Cas9 has been implicated in virulence, specifically host cell adhesion (Sampson et al. 2013, Capel et al. 2016, Heidrich et al. 2019), but the precise mechanisms remain unclear.

scaRNAs are a novel class of noncoding RNAs genetically linked to type II CRISPR/Cas loci in various pathogenic bacteria, including *N. meningitidis* (Sampson et al. 2013, 2019, Guzina et al. 2018, Ratner et al. 2019, Markle et al. 2021) (Fig. S1). Aside from *F. novicida*, their functional role in bacterial pathogenesis has not yet been experimentally assessed. In *N. meningitidis*, scaRNA (previously described as NMnc0040; Heidrich et al. 2017) was shown to bind to Nme1Cas9 (Heidrich et al. 2019) but neither to Hfq (Heidrich et al. 2017) nor to ProQ (Bauriedl et al. 2020). Although it has been shown to be constitutively expressed when bacteria are grown in rich medium, its function, if any, remains unknown.

Using *N. meningitidis* as a model system, we sought to investigate how scaRNA and Nme1Cas9 impact bacterial fitness under infection-relevant conditions—particularly oxidative stress and iron limitation. Mechanisms for coping with oxidative stress are crucial for mucosal pathogens like *N. meningitidis* (Criss and Seifert 2012). Reactive oxygen species (ROS), including superoxide (O₂⁻), hydrogen peroxide (H₂O₂), and hydroxyl radicals (HO·), arise during aerobic respiration and immune responses, causing damage to DNA, lipids, and proteins (Lo et al. 2009). Under aerobic conditions, the toxicity of H₂O₂ is exacerbated by iron via the Fenton reaction—which also catalyzes the formation of harmful hydroxyl radicals when excess iron is present (Cornelissen 2018, Tang et al. 2021, Murdoch and Skaar 2022)—necessitating well‐regulated systems to sense and manage intracellular iron and ROS levels (Cornelissen 2018, Murdoch and Skaar 2022). In the human body, iron is a critical yet tightly regulated nutrient for bacterial growth, with high‐affinity uptake systems playing a central role in meningococcal iron homeostasis (Yadav et al. 2019, Murdoch and Skaar 2022). Conversely, iron deficiency is predicted to increase bacterial susceptibility to phage infection (Muratore and Weitz 2021). Consequently, intracellular iron availability is governed by a complex regulatory process comprising transcriptional, translational, and posttranslational mechanisms that control the expression of proteins and noncoding RNAs (Charbonnier et al. 2022, Papenfort and Melamed 2023).

Here, we show that Nme1Cas9 expression is induced under iron-limiting and oxidative stress conditions mediated by H₂O₂ exposure. Deletion of *cas9* and *scaRNA* leads to the upregulation of ∼50 proteins involved in ion transport, energy production, and amino acid metabolism, alongside the downregulation of protein translation genes. We identify the *efeUOB* operon, which encodes a ferrous iron transport system (Grifantini et al. 2003, Cao et al. 2007), as a target of Nme1Cas9 repression. Furthermore, we demonstrate that scaRNA binds to the translational start site of *efeO* mRNA *in vitro*, and together with Nme1Cas9 reduces the stability and translation of *efeUOB* mRNA *in vivo*. Together, our findings reveal a novel mechanism by which the meningococcal CRISPR/Cas system regulates bacterial metabolism and stress responses under infection-relevant conditions.

## Materials and methods

### Bacterial strains and growth conditions

*Neisseria meningitidis* cells were grown at 37°C and 5% CO_2_ on either Columbia blood agar plates (COS) or GC medium base (GCB) agar plates [22.2 mM glucose, 0.68 mM glutamine, 0.45 mM cocarboxylase, and 1.23 mM Fe(NO_3_)_3_]. GCB agar plates were further supplemented with 50 µg/ml kanamycin, 20 µg/ml chloramphenicol or 20 µg/ml erythromycin as required. Liquid cultures were grown either in GCB liquid medium supplemented with sodium bicarbonate and ferric nitrate (GCBL^++^) or in Eagle’s minimal essential medium supplemented with 10% fetal calf serum, 1% nonessential amino acids, and 1% sodium pyruvate (EMEM^+++^) at 37°C and 200 rpm. The optical density (OD) at 600 nm (OD_600nm_) was monitored hourly for growth-curve analysis.

For bacterial growth under different environmental stress conditions, meningococci were grown to mid-logarithmic phase in GCBL^++^ and then exposed to the following stress conditions for 10 min: (a) control (no stress), (b) iron limitation by addition of 250 µM 2,2’-dipyridil (Fagnocchi et al. 2015), (c) oxidative stress by addition of 135 µM H_2_O_2_ (Fagnocchi et al. 2015), (d) oxidative stress by addition of 2 mM paraquat (Takahashi et al. 2018), (e) osmotic stress by addition of 5 M NaCl (Fantappie et al. 2009), and (f) membrane stress by addition of 0.01% sodium dodecyl sulfate (SDS) (Fantappie et al. 2009). Details on the number of replicates and the statistical analyses are provided alongside the respective data.

*Escherichia coli* strains were grown at 37°C on Luria–Bertani (LB) agar plates supplemented with 30 µg/ml chloramphenicol or 50 µg/ml erythromycin, as appropriate. Liquid cultures were grown in LB medium supplemented with the appropriate antibiotic. The bacterial suspension was incubated at 37°C and shaken at 200 rpm.

### Construction of *N. meningitidis* mutant strains

A list of bacterial strains is given in Table S1 and a list containing all plasmids used for mutant construction is given in Table S2. Table S3 provides a complete list of all DNA oligonucleotides used for mutant construction.

*Neisseria meningitidis* 8013 Δ*cas9*, ΔtracrRNA, ΔscaRNA, Δ*cas9 cas9*^+^, and ΔtracrRNA tracrRNA^+^ mutant strains were constructed as previously described (Zhang et al. 2013, Heidrich et al. 2019).

The scaRNA complementation and overexpression strains in *N. meningitidis* 8013 were constructed by using the neisserial complementation plasmid pMR68 (Ramsey et al. 2012). For scaRNA complementation, the nucleotide sequence of scaRNA together with its native promoter sequence was amplified from genomic DNA (gDNA) by polymerase chain reaction (PCR) using primer pairs 1499/1500 and cloned into the *EcoRV*/*SalI* predigested plasmid pMR68. The resulting plasmid was transformed into the *trpB*/*iga* gene locus of the ΔscaRNA strain via natural transformation. Erythromycin-resistant transformants were verified by colony PCR and gDNA sequencing.

The scaRNA overexpression strains were constructed similarly, except for one difference. The nucleotide sequence of the scaRNA (without its native promoter) was amplified by PCR from gDNA using primer pairs 1520/1500 and cloned directly after the -10-box of the tetracycline-inducible promoter of the *EcoRV*/*SalI* predigested plasmid pMR68 (Fig. S1B). For the induction of the promoter, 2 ng/ml anhydrotetracycline (ATc) was added to a liquid culture.

### Hydrogen peroxide and iron limitation sensitivity assay

The hydrogen peroxide assay was performed as previously described with some minor modifications (Grifantini et al. 2004). Briefly, *N. meningitidis* strains were grown overnight at 37°C and 5% CO_2_ on 5% Columbia blood agar plates. The strains were adjusted to an OD_600nm_ of 0.2 in GCBL^++^ medium (supplemented with 1.23 mM Fe(NO_3_)_3_) or GCBL^++^ medium supplemented with 500 µM 2,2-dipyridil (iron depletion) and then exposed to 200 µM H_2_O_2_. Bacteria were cultured in 96-well microtiter plates at 37°C and vigorous shaking. Growth was monitored for 18 h using the Infinite F 200 Pro Instrument (Tecan). The optical density at 620 nm (OD_620nm_) was measured every 30 min. Details on the number of replicates and the statistical analyses are provided alongside the respective data.

### RNA extraction for northern blot analysis

*Neisseria meningitidis* strains were grown to mid-logarithmic growth phase (OD_600nm_ ∼ 0.5) in EMEM^+++^ medium at 37°C and 200 rpm. The suspension was pelleted at 4000 rpm for 10 min at 4°C, shock frozen in liquid nitrogen, and stored at −80°C until RNA extraction. The frozen pellets were thawed on ice and lysed by resuspending the cells in 600 µl 0.5 mg/ml lysozyme in 1xTE buffer (pH 8.0), 60 µl 10% SDS, and 66 µl 3 M sodium acetate (pH 5.2) followed by an incubation at 64°C for 2 min. Total RNA was isolated using the hot phenol method (Blomberg et al. 1990).

### Northern blot analysis

Northern blot analysis was performed using either DIG-labelled DNA probes or γ^32^P-ATP end-labelled DNA oligonucleotides.

For nonradioactive northern blot analysis, 10 µg of total RNA was loaded onto a 1% formaldehyde agarose gel supplemented with 0.02% GelRed® (Biotium) in 1x MOPS (40.5 g/l MOPS, 4.1 g/l sodium acetate, 10 mM ethylenediaminetetraacetic acid (EDTA), pH 8.0, and 40 mM sodium hydroxide) at 80 V for 2–3 h. The RNA was then transferred onto a positively charged Hybond membrane (GE Healthcare) overnight by capillary transfer using 20x SSC (175.5 g/l sodium chloride, 88.2 g/l sodium citrate) as transfer buffer. After UV cross-linking, the membrane was hybridized with DIG-labelled DNA probes overnight at 68°C. For nonradioactive labelling of a northern blot probe, a specific DNA fragment was amplified by PCR. The product was mixed with 1x hexanucleotide mix (Roche), 1x DIG DNA labelling mix (Roche) and 2 U Klenow enzyme (Roche) and incubated overnight at 37°C. A 50 ng/ml DIG-labelled DNA probe was prepared in hybridization high SDS buffer.

For radioactive northern blot analysis, 5 µg of total RNA sample was separated on a 6% polyacrylamide (PAA) gel supplemented with 8.3 M urea at 300 V for 2–3 h. After electroblotting to a positively charged Hybond-XL membrane (GE Healthcare), the RNA was UV cross-linked to the membrane and hybridized with a γ^32^P-ATP end-labelled DNA oligonucleotide at 42°C overnight. To label a radioactive northern blot probe, 1 pmol of an oligonucleotide was mixed with 1x kinase buffer, 10 U T4 PNK, and 20 µCi γ^32^P-ATP and incubated at 37°C for 1 h. Then, the reaction was purified using the Microspin G-25 columns (GE Healthcare) according to the manufacturer’s instructions.

Details on the number of replicates and the statistical analyses are provided alongside the respective data.

### Sample preparation for quantitative proteomics

Bacterial cells were grown in EMEM^+++^ medium to an OD_600nm_ of 0.5. Cells were pelleted and lysed in 100 µl lysis buffer (20 mM Tris–HCl, pH 7.5, 150 mM KCl, 1 mM MgCl_2_, 0.2% Triton X-100, 1 mM dithiothreitol (DTT), 1 mM PMSF, 40 U DNase I, and RNase) using 0.1 mm glass beads by vortexing 10 times for 30 s. The lysates were centrifuged, and the supernatants were mixed with an equal volume of 5x protein loading buffer.

Protein precipitation was performed overnight at −20°C with four volumes of acetone. Proteins were dissolved in NuPAGE® LDS sample buffer (Life Technologies), reduced with 50 mM DTT at 70°C for 10 min and alkylated with 120 mM iodoacetamide at room temperature for 20 min. Separation was performed on NuPAGE® Novex® 4%–12% Bis–Tris gels (Life Technologies) with MOPS buffer according to the manufacturer’s instructions. The gels were washed with water three times for 5 min and stained with Simply Blue™ Safe Stain (Life Technologies) for 45 min. After washing with water for 2 h, each gel lane was cut into 15 slices. The excised gel bands were destained with 30% acetonitrile in 0.1 M NH_4_HCO_3_ (pH 8.0), shrunk with 100% acetonitrile, and dried in a vacuum concentrator (Concentrator 5301, Eppendorf, Germany). Digests were performed overnight at 37°C in 0.1 M NH_4_HCO_3_ (pH 8.0) with 0.1 µg trypsin per gel band. After removal of the supernatant, peptides were extracted from the gel bands with 5% formic acid and the extracted peptides were pooled with the supernatant.

### MS/MS analysis

Nanoflow liquid chromatography coupled to tandem mass spectrometry (nanoLC–MS/MS) analyses were performed on an LTQ-Orbitrap Velos Pro (Thermo Scientific) equipped with a PicoView ion Source (New Objective) and coupled to an EASY-nLC 1000 (Thermo Scientific). Peptides were loaded on capillary columns (PicoFrit, 30 cm × 150 µm ID, New Objective) self-packed with ReproSil-Pur 120 C18-AQ, 1.9 µm (Dr. Maisch) and separated with a 30-min linear gradient from 3% to 30% acetonitrile and 0.1% formic acid and a flow rate of 500 nl/min.

MS scans were acquired in the Orbitrap analyzer with a resolution of 30 000 at m/z 400. MS/MS scans were acquired in the Orbitrap analyzer with a resolution of 7500 at m/z 400 using HCD fragmentation with 30% normalized collision energy. A TOP5 data-dependent MS/MS method was used; dynamic exclusion was applied with a repeat count of 1 and an exclusion duration of 30 s; singly charged precursors were excluded from selection. The minimum signal threshold for precursor selection was set to 50 000. Predictive AGC was used with an AGC target value of 1 × 10^6^ for MS scans and 5 × 10^4^ for MS/MS scans. The Lock Mass option was used for internal calibration in all runs using background ions from protonated decamethylcyclopentasiloxane (m/z 371.10124).

### MS data analysis

Raw MS data files were analysed using MaxQuant version 1.6.2.2 (Cox and Mann 2008). Database searches were performed using Andromeda, which is included in the version of MaxQuant used. Searches were performed against the NCBI *N. meningitidis* strain 8013 database. In addition, a database of common contaminants was used. The search was performed with tryptic cleavage specificity with three miscleavages allowed. Protein identification was performed under false discovery rate (FDR) control of 1% at protein and peptide level. In addition to the MaxQuant default settings, the search was performed against the following variable modifications: protein N-terminal acetylation, Gln to pyro-Glu formation (N-term. Gln), and oxidation (Met). Carbamidomethyl (Cys) was set as the fixed modification. log_2_-transformed label-free quantitation (LFQ) intensities were used for protein quantification (Cox et al. 2014). Proteins with less than two identified razor/unique peptides were discarded. Further data analysis was performed using R scripts developed in-house. LFQ intensities were used and missing LFQ intensities were imputed with values close to baseline. Data imputation was performed using values from a standard normal distribution with a mean of the 5% quantile of the combined log10-transformed LFQ intensities and a standard deviation of 0.1. To identify significantly enriched proteins, the median log2 transformed protein ratio was calculated from the three replicate experiments and boxplot outliers were identified in intensity bins of at least 300 proteins. Sample versus control log2 transformed protein ratios with values outside a 3x interquartile range (IQR) were considered significantly enriched. The mass spectrometry proteomics data have been deposited with the ProteomeXchange Consortium via the PRIDE (Perez-Riverol et al. 2022) partner repository with the dataset identifier PXD037567.

### Gene set analysis of proteomics data incorporating directionality of protein expression

To identify differentially regulated sets of functionally related proteins we used the COG functional classification of proteins (Tatusov et al. 2001, Galperin et al. 2015) together with the log_2_-fold changes (log_2_ f.c.) and the adjusted *P*-values for each protein obtained using the R package limma (Ritchie et al. 2015) as input for gene set analyses using the R package Piano v.1.18.1 (Väremo et al. 2013). In this approach, significance estimation is performed after functional classification of differentially expressed proteins and applied to whole protein sets. In contrast to the classical protein-based approach, this approach is more suitable for detecting differences in the proteome when the expression differences of individual proteins are small but affect e.g. entire metabolic and/or regulatory pathways. As described in Väremo et al. (2013), the analyses were repeated using different statistical methods (‘*P*-value’, ‘Wilcoxon’, ‘Fisher’, ‘Stouffer’, ‘Reporter’, and ‘Tail Strength’) for significance estimation together with the consensus scoring approach. For the interpretation of the results, three different directionality classes were considered, as recommended in Väremo et al. (2013). The nondirectional class can be interpreted as affected by differential expression in general. For the mixed-directional class, a gene set may be significantly affected by differentially expressed genes in either or both directions. Here, a gene set can be significantly affected by both up- and downregulation if it contains two subsets of genes that are coordinately regulated in opposite directions. Finally, the distinct-directional class aims to identify gene sets that are significantly affected by regulation in a distinct direction.

### Rifampicin stability assay

Bacterial cells were grown to an OD_600nm_ of 0.5 and treated with 500 µg/ml rifampicin in DMSO. At specific time points (0, 2, 8, 16, and 32 min), RNA samples were taken from the culture, immediately mixed with 1.5 ml stop solution (95% ethanol, 5% phenol), and shock frozen in liquid nitrogen. RNA was isolated using the hot phenol method (Blomberg et al. 1990). Total RNA was analysed by northern blot analysis and RNA expression was quantified using ImageJ (Schneider et al. 2012). Details on the number of replicates and the statistical analyses are provided alongside the respective data.

### *In vitro* T7 RNA transcription (IVT) and 5′ end labelling

For *in vitro* RNA synthesis, a DNA template was generated either by Q5-PCR or by annealing oligonucleotides carrying a T7 promoter sequence in annealing buffer (100 mM Tris–HCl, pH 7.5, 1 M sodium chloride, 10 mM EDTA). The template DNA was transcribed using the AmpliScribe T7-Flash Transcription Kit (Biozym). The *in vitro* transcribed RNA was gel purified on a 10% PAA gel supplemented with 7 M urea. Gel slices containing the RNA of interest were excised and the RNA was eluted in elution buffer (100 mM sodium acetate, 0.1% SDS, 10 mM EDTA, pH 8.0) overnight at 4°C with agitation. The next day, the RNA was recovered by P:C:I extraction and precipitated by adding three volumes of a 30:1 mixture (ethanol, 3 M sodium acetate).

First, 20 pmol of *in vitro* transcribed RNA was dephosphorylated by a quick CIP treatment (NEB). After P:C:I extraction and RNA precipitation, 20 pmol RNA was incubated with 20 µCi γ-^32^P-ATP and 1 U T4 polynucleotide kinase (PNK) in 1x kinase buffer. Unbound phosphates were removed by purification on Microspin G-50 columns (GE Healthcare). The labelled RNA was then gel purified on a 10% PAA gel supplemented with 8.3 M urea as described above.

### Purification of Nme1Cas9 protein

For recombinant expression, the full-length wild-type Nme1Cas9 ORF (WP_014574210) was PCR-amplified from genomic *N. meningitidis* DNA and cloned via SLIC (Jeong et al. 2012) into a *NcoI*-opened pETM-41 vector. The final construct contained an N-terminal, TEV-cleavable His-MBP tag. The pETM-41 expression construct was transformed into *E. coli* Rosetta™ 2 (DE3) cells (Novagen) and positive clones were selected. The expression culture was inoculated from an overnight culture and incubated at 37°C and 200 rpm. When the culture reached an OD_600nm_ of 0.5, expression was induced by adding isopropyl β-D-1-thiogalactopyranoside (IPTG) to a final concentration of 0.2 mM and temperature was lowered to 18°C for overnight expression. After expression, cells were harvested (15 min at 6000 × *g* and 4°C), snap frozen in liquid nitrogen and stored at −20°C. All purification steps were performed either on ice, in a cold room, or in a refrigerated cabinet. The cell pellet was thawed and resuspended in lysis buffer [10 ml/g cell pellet; 500 mM NaCl, 40 mM Tris–HCl pH 8.0, 0.5 mM TCEP, 20 mM imidazole, and 10% (v/v) glycerol] in the presence of cOmplete™ EDTA-free protease inhibitor cocktail (Roche). Cells were lysed by sonication (four 1-min pulses at maximum intensity, on ice; LABSONIC©P sonifier; B. Braun Biotech International). The lysate was clarified by centrifugation (30 min at 30 000 × *g*, 4°C). The clarified lysate was incubated with Ni-NTA agarose (Qiagen; equilibrated with lysis buffer) for 1 h before passing through a glass chromatography column (Bio-Rad). The flow was collected, and the column bed was washed twice with 10 column volumes of lysis buffer, 10 column volumes of IMAC wash buffer [1 M NaCl, 40 mM Tris–HCl pH 8.0, 0.5 mM TCEP, 20 mM imidazole, and 10% (v/v) glycerol] and 10 column volumes of lysis buffer. For elution, the column was washed six times with one column volume of IMAC elution buffer [250 mM NaCl, 20 mM Tris–HCl pH 8.0, 0.5 mM TCEP, 500 mM imidazole, and 10% (v/v) glycerol]. Elution fractions containing the His–MBP–Nme1Cas9 fusion protein were pooled and dialysed overnight against dialysis buffer [300 mM KCl, 20 mM HEPES–NaOH pH 7.5, 1 mM TCEP, and 10% (v/v) glycerol] in the absence or presence of TEV protease to either retain the intact His–MBP–Nme1Cas9 fusion protein or to obtain untagged Nme1Cas9 protein. Dialysates were applied to a HiTrap SP HP cation exchange column (Cytiva), equilibrated with dialysis buffer, the column was washed with 20 column volumes of dialysis buffer, and the protein was finally eluted with a linear gradient to 1 M KCl over 20 column volumes. IEX eluates containing Nme1Cas9 protein were pooled and either only concentrated and rebuffered to dialysis buffer using Centricon® concentrators (Merck) or concentrated and rebuffered by size exclusion chromatography on a HiLoad Superdex200 16/600 pg column (Cytiva), if the sample required further purification. Final samples were concentrated up to 0.5 mg/ml, frozen in liquid nitrogen and stored at −80°C.

### Electrophoretic mobility shift assay

*In vitro* binding reactions were carried out as described previously (Rousseau et al. 2018). Briefly, 4 nM 5′ end labelled RNA was mixed with increasing amounts of purified Nme1Cas9 protein or unlabelled RNA in 1x binding buffer (20 mM HEPES, pH 7.5, 50 mM KCl, 0.1 mM EDTA, 0.5 mM DTT, and 30 µg/ml heparin). The binding reactions were carried out at 37°C for 30 min. After the addition of 1x native loading dye (0.5 × TBE, 50% glycerol, 0.2% xylene cyanol, and 0.2% bromophenol blue), the samples were analysed on an 8% native PAA gel in 1x TBE buffer.

### In-line probing assay

In-line probing assays were performed for structural and interaction site analysis. For sample preparation, 4 nM ^32^P-labelled scaRNA was mixed with 0 nM, 10 nM, 100 nM, or 1 µM unlabelled mRNA in 1x in-line reaction buffer (100 mM Tris–HCl, pH 8.3, 40 mM MgCl_2_, and 200 mM KCl) and incubated for 40 h at room temperature. For the RNase T1 ladder, 0.4 pmol of 5′-end labelled scaRNA in 1x sequencing buffer (Ambion) was denatured at 95°C for 1 min, cooled to 37°C on ice, and then incubated with 0.1 U RNase T1 (Ambion) at 37°C for 5 min. For the OH ladder, 0.4 pmol of 5′-end labelled scaRNA was incubated in 1x alkaline hydrolysis buffer (Ambion) at 95°C for 5 min. Reactions were stopped by adding an equal volume of colourless gel loading solution (10 M urea, 1.5 mM EDTA, and pH 8.0) and analysed on a 6% PAA gel. Electrophoresis was performed in 1x TBE buffer at 40 W for 3 h.

### *In vitro* cleavage assay

*In vitro* cleavage reactions were performed as described previously (Rousseau et al. 2018). Briefly, 4 nM 5′-end labelled RNA was mixed with increasing amounts of purified Nme1Cas9 protein in 1x cleavage buffer (20 mM HEPES, pH 7.5, 50 mM KCl, 0.1 mM EDTA, 0.5 mM DTT, and 10 mM MgCl2). The reactions were incubated for 30 min at 37°C. The samples were analysed on a 10% denaturing PAA gel. Alternatively, 4 nM of 32P-labelled scaRNA was incubated in the absence or presence of 150 nM purified Nme1Cas9 protein in 1x structure buffer (10 mM Tris–HCl, pH 7.0, 0.1 M KCl, and 10 mM MgCl2) and incubated at 37°C for 15 min. The reactions were analysed on an 8% PAA gel in 1x TBE buffer.

### Quantification of gene expression by RT-qPCR

First, 2 µg of DNA-free RNA was reverse transcribed into cDNA using SuperScript™ II Reverse Transcriptase (Invitrogen) according to the manufacturer’s instructions. For the quantitative PCR (qPCR) reaction, ∼5 ng/µl of cDNA was mixed with 20 µM of each oligonucleotide in SYBR Green 2x Master Mix (Thermo Fisher Scientific). The reactions were pipetted into a 96-well plate and each cDNA was run in triplicate. qPCR was performed using a StepOnePlus system and comparative threshold cycle (ΔΔC_T_) analysis (Livak and Schmittgen 2001) was used. The 16S rRNA housekeeping gene was used as an endogenous control to normalise gene expression. Details on the number of replicates and the statistical analyses are provided alongside the respective data.

### Western blot analysis

For protein expression analysis, *N. meningitidis* was grown in GCBL^++^ medium to an OD_600nm_ of 0.5. The suspension was pelleted and resuspended in 5x protein loading buffer (300 mM Tris–HCl, pH 6.8, 0.2% bromophenol blue, 50% glycerol, 77 g/l DTT, and 100 g/l SDS). After incubation at 95°C for 5 min, 0.01 OD_600nm_ equivalents of the sample were analysed on a 12% SDS–PAA gel. Proteins were transferred to a PVDF membrane (GE Healthcare) by electroblotting at 100 V and 4°C for 1 h in 1x transfer buffer (10% methanol, 30.25 g/l Tris base, and 150 g/l glycine). For GFP detection, a mouse monoclonal antibody (1:3000) was used as the primary antibody, followed by a horseradish peroxidase (HRP)-conjugated anti-mouse antibody (1:10 000). GroEL was used as a loading control. For GroEL detection, a rabbit monoclonal antibody (1:10 000) was used as the primary antibody, followed by an HRP-conjugated anti-rabbit antibody (1:10 000). Western blot was developed using Pierce™ ECL Western Blotting Substrate (Thermo Fisher Scientific) according to the manufacturer’s instructions. Signal was detected using the ChemiDoc MP imaging system (Bio-Rad) and protein expression was quantified using ImageJ (Schneider et al. 2012). Details on the number of replicates and the statistical analyses are provided alongside the respective data.

### Structural and RNA–RNA interaction site analysis

The UNAFold web server (Zuker 2003) was used to predict RNA secondary structures. RNAhybrid, IntraRNA, CopraRNA, and BLASTN (Altschul et al. 1990, Rehmsmeier et al. 2004, Wright et al. 2014, Mann et al. 2017) were used to predict potential interaction sites between two RNAs.

### Sequence availability

The BLASTN database (Nucleotide Basic Local Alignment Search Tool, https://blast.ncbi.nlm.nih.gov/Blast.cgi?PAGE_TYPE=BlastSearch) was used to obtain information on meningococcal sequences. The meningococcal strains and their accession numbers are listed below*: N. meningitidis* 8013 (NC_017501.1).

## Results

### scaRNA and Nme1Cas9 show opposite expression patterns in response to external stress

**Figure 1:**
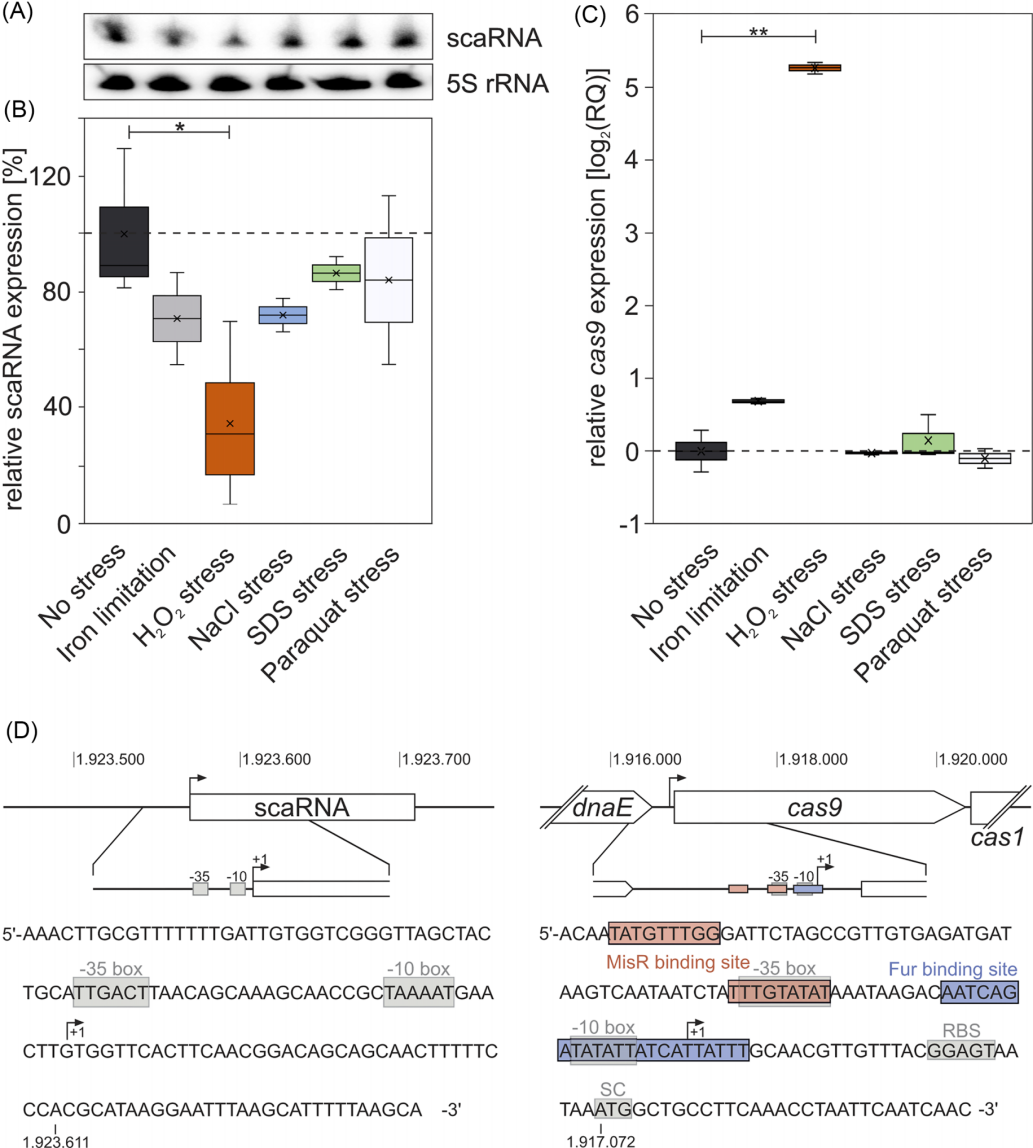
Expression analysis of scaRNA and *cas9* under different stress conditions. (A) Northern blot analysis of total RNA extracted in the mid-logarithmic growth phase for scaRNA expression. The housekeeping gene 5S rRNA served as a loading control. (B) Quantification of scaRNA expression as compared to the unstressed wild-type (scaRNA expression level was set to 100%, dashed line). *n* = 3. (C) Relative quantification (RQ) of *cas9* mRNA expression changes in the wild-type strain under different stress conditions compared to unstressed (no stress) *cas9* expression using RT-qPCR. Data in (B) and (C) are shown as box-and-whisker plots from three independent experiments. The colored boxes represent the IQR and are divided by a horizontal line indicating the median. A cross marks the mean, and the whiskers indicate the minimum and maximum values. *P*-values were calculated using a two-tailed Welch’s *t-*test comparing each stress condition with the unstressed condition. *: *P*-value < .05, **: *P*-value < .01. *n* = 3. (D) Schematic representation of the scaRNA and *cas9* promoter regions. The ribosomal binding site (RBS), the start codon (SC), and the −10 and the −35 boxes are shown in grey; potential MisR and Fur binding sites are shown in red and blue, respectively.

In order to analyse the expression of scaRNA and *cas9* in response to conditions that are relevant during the processes of colonisation and/or infection, the *N. meningitidis* wild-type strain 8013 was cultivated to the mid-logarithmic growth phase in EMEM^+++^ medium and subjected to different stress conditions *in vitro*. These included (i) osmotic stress induced by 5 M NaCl (Fantappie et al. 2009), (ii) envelope stress induced via 0.01% SDS (Fantappie et al. 2009), (iii) iron limitation by supplementing 500 µM 2,2-dipyridil (Fagnocchi et al. 2015), (iv) oxidative stress induced by exposure to 135 µM H_2_O_2_ (Fagnocchi et al. 2015), or (v) 2 mM paraquat (Takahashi et al. 2018) (Fig. 1). Hydrogen peroxide can freely diffuse across the inner and outer membrane of Gram-negative bacteria and is converted into highly reactive hydroxyl radicals by metal ions, such as Fe²^+^ via the Fenton reaction (Cornelis et al. 2011, Bradley et al. 2020). Paraquat mainly produces intracellular superoxide ($\dot O_2^ - $$)$, which can lead to intracellular H_2_O_2_ generation and hydroxyl radicals in several steps (Hassan and Fridovich 1979, Korshunov and Imlay 2002).

As illustrated in Fig. 1(A) and (B) , northern blot analysis showed scaRNA expression to be markedly reduced in response to H_2_O_2_ and to a lesser extent to iron limitation, but not to osmotic stress, membrane stress, or paraquat exposure. RT-qPCR analyses demonstrated that *cas9* was significantly upregulated in response to hydrogen peroxide and to a lesser extent to iron limitation (Fig. 1C). Surprisingly, the expression of scaRNA and Nme1Cas9 showed opposite expression patterns particularly in response to H₂O₂ and iron limitation. Therefore, while H₂O₂ decreases the concentration of scaRNA, it strongly increases the concentration of Nme1Cas9. The expression data prompted us to computationally screen the 5′ upstream regions of both genes for binding motifs of the ferric iron uptake regulator (Fur) using FIMO (Grant et al. 2011), which primarily functions as a transcriptional repressor in the presence of ferric iron (Grifantini et al. 2003, Delany et al. 2006). Additionally, we screened for binding motifs of the transcription factor MisR, which is known to function as an activator in the presence of Fur repression in response to oxidative stress (Tzeng et al. 2008).

As shown in Fig. 1(D), the *in silico* analyses indicated the existence of a Fur binding site at the −10 box, in addition to a MisR binding site situated upstream of the −35 box and a second Fur binding site at the −35 box within the *cas9* 5′ upstream region. The location of the Fur binding site indicates that Fur is predicted to act as a transcriptional repressor of *cas9* expression in the presence of ferrous iron. Indeed, iron depletion results in the de-repression of *cas9* (Fig. 1C). Likewise, in line with the experimental data and the location of the MisR binding sites, MisR is expected to activate *cas9* expression in response to oxidative stress (Tzeng et al. 2008). It is notable that no such binding sites could be detected in the promoter region of scaRNA (Fig. 1D), which suggests that scaRNA expression might be regulated at the posttranscriptional level, i.e. via RNA stability.

Considering the opposite expression patterns in response to external stress, we proceeded to investigate whether the deletion of scaRNA and/or Nme1Cas9 affected the fitness of the bacteria under infection-relevant conditions *in vitro* in response to the presence of H₂O₂ and iron limitation.

### The nonessential scaRNA together with Nme1Cas9 reduces meningococcal resistance to oxidative stress

We first assessed whether scaRNA *per se* affected meningococcal growth in (rich) EMEM^+++^ medium *in vitro* using scaRNA deletion or overexpression strains. This confirmed our previous observations that neither deletion nor overexpression of scaRNA impaired the growth of *N. meningitidis* under nutrient replete conditions (Fig. S2) (Heidrich et al. 2017). Minor variations in OD_600nm_ slopes were within experimental variability and did not indicate significant growth-rate differences under the tested conditions.

**Figure 2:**
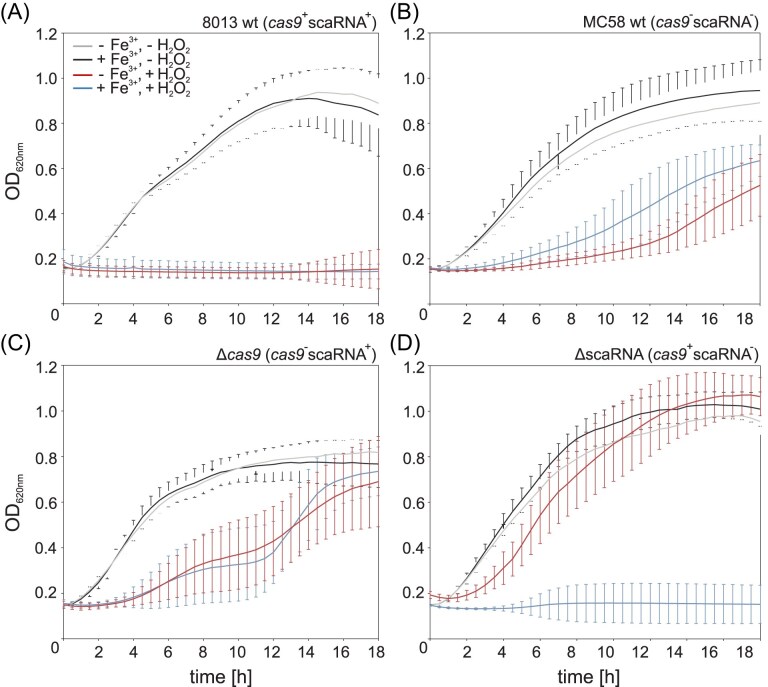
Growth analysis of *N. meningitidis* 8013 wild-type (A), Δ*cas9* (C), ΔscaRNA (D), and *N. meningitidis* MC58 wild-type (B) strains with and without oxidative stress and/or iron limitation as indicated. The optical density was determined at 620 nm once every 30 min for 18 h. The time in hours is displayed on the *x*-axis and the OD at 620 nm on the *y*-axis. The data represent mean values of five independent experiments and the standard error of the mean is depicted for each strain and time point.

Because all attempts to generate a Δ*cas9* ΔscaRNA double mutant in strain 8013 were unsuccessful, we additionally included strain MC58, which naturally lacks the entire CRISPR/Cas locus, as a CRISPR/Cas-locus-negative reference background in the oxidative-stress assays shown in Fig. 2. Because MC58 is not isogenic to strain 8013, it is used for contextual comparison only and not as a genetic substitute for a constructed double mutant. Any mechanistic inference is based on the isogenic 8013 mutants and the defined reporter system. As further illustrated in Fig. 2 and Fig. S3, all strains exhibited comparable growth in the absence of H_2_O_2_ irrespective of their genotype or the availability of iron in the medium (Fig. S3A and B). In the presence of H_2_O_2_, strains lacking *cas9* (*cas9*^−^) were observed to grow, irrespective of the availability of iron in the medium or the presence of scaRNA (Fig. 2B and C), Fig. S3C and D). This indicates that the presence of *cas9* itself impairs resistance against oxidative stress in *N. meningitidis*. In the presence of H_2_O_2_, only strain *cas9^+^scaRNA*^−^ was able to grow under iron-depleted conditions whereas iron abolished growth in this mutant (Fig. 2A and D). This indicates that in the presence of H_2_O_2_ and *cas9*, scaRNA affected bacterial fitness in an iron-dependent manner.

Therefore, (i) Nme1Cas9 and scaRNA exert distinct effects on bacterial growth in response to H_2_O_2_, and (ii) scaRNA contributes to iron-dependent oxidative-stress adaptation in the presence of Nme1Cas9. Accordingly, the next objective was to determine whether the deletion of scaRNA or Nme1Cas9 resulted in any alterations in protein expression that could be responsible for the observed phenotypes employing a genome-wide proteomics approach.

### Nme1Cas9 and scaRNA have an overlapping yet distinct regulatory impact on the meningococcal proteome

**Figure 3:**
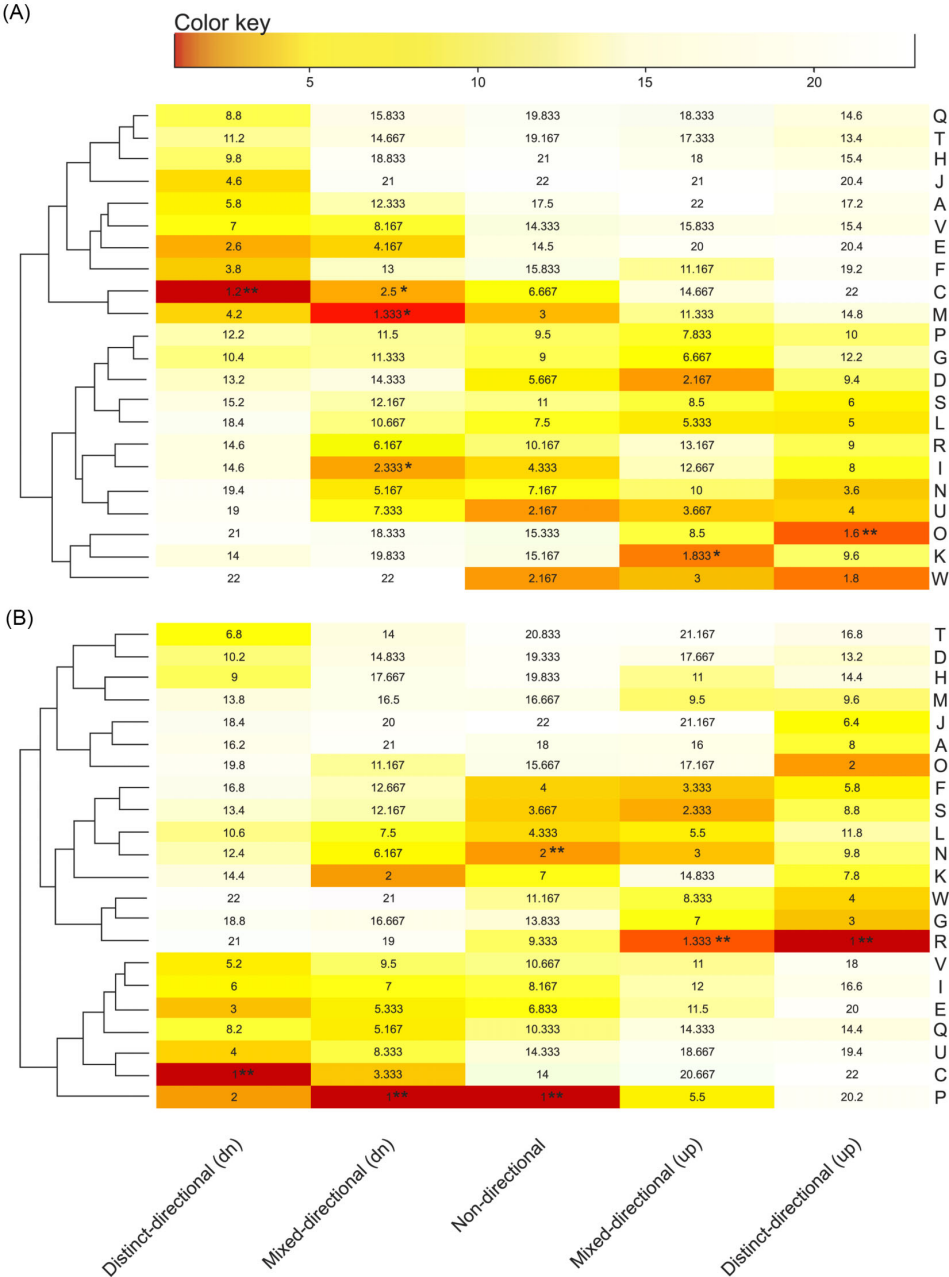
Heat map of consensus scores and hierarchical clustering of COG functional classes of (A) Δ*cas9* and (B) ΔscaRNA proteomes compared to the wild-type proteome obtained by Piano v.1.18.1 (Väremo et al. 2013). Consensus scores are colour coded and asterisks indicate whether a given functional class (indicated on the right side of each heat map) is significantly differentially expressed in the manner indicated at the bottom of the lower heat map. For the interpretation of the results, three different directionality classes were considered. The nondirectional class can be affected by differential expression in general. For the mixed-directional class, a gene set may be significantly affected by differentially expressed genes in either or both directions. Finally, the distinct-directional class aims to identify gene sets that are significantly affected by regulation in a distinct direction. **: FDR < 0.01, *: FDR < 0.05. COG (cluster of orthologous genes) functional categories (Galperin et al. 2015, Tatusov et al. 2001): A, RNA processing and modification, C, energy production and conversion; D, cell cycle control, mitosis, and meiosis; E, amino acid transport and metabolism; F, nucleotide transport and metabolism; G, carbohydrate transport and metabolism; H, coenzyme transport and metabolism; I, lipid transport and metabolism; J, translation; K, transcription; L, replication, recombination, and repair; M, cell wall/membrane biogenesis; N, cell motility; O, posttranslational modification, protein turnover, and chaperones; P, inorganic ion transport and metabolism; Q, secondary metabolite biosynthesis, transport, and catabolism; R, general function prediction only; S, function unknown; T, signal transduction mechanisms; U, intracellular trafficking and secretion; V, defense mechanisms; and W, extracellular structures.

We used peptide quantitative mass spectrometry to assess gene expression differences between Δ*cas9* and ΔscaRNA mutants and the wild-type strain. As the presence of H_2_O_2_ had been demonstrated to negatively affect bacterial viability (see Fig. 2 and Fig. S3), the wild-type, Δ*cas9* and ΔscaRNA strains were cultivated in plain EMEM^+++^ medium until they reached the mid-logarithmic growth phase. Because genes interact in pathways that can be grouped into functional classes (Tatusov et al. 2001, Galperin et al. 2015), even minor yet coordinated expression changes across members of the same pathway can have substantial biological implications (Subramanian et al. 2005). Therefore, we complemented single-gene proteomic analyses with gene set analysis to detect differential expression of entire functional groups (Väremo et al. 2013). Figure 3 summarizes pathway-level effects derived from gene-set enrichment, whereas Fig. S4 highlights individual proteins with significant differential expression.

Consistent with the phenotypic data, *cas9* and scaRNA deletion mutants exhibited downregulation of proteins associated with energy production (COG category C), including those involved in glutamate metabolism, which forms part of the oxidative stress response in *N. meningitidis* (Schoen et al. 2014), which is important for both colonization and invasive disease (Pagliarulo et al. 2004, Ampattu et al. 2017).

Proteins involved in posttranslational modification, protein turnover, and chaperones (COG category O) or transcription (COG K) exhibited an upregulation exclusively in the *Δcas9* mutant relative to the wild-type strain. Similarly, proteins involved in cell wall/membrane biogenesis (COG M) and lipid transport and metabolism (COG I) exhibited a downregulation exclusively in the *Δcas9* mutant.

On the other hand, proteins involved in inorganic ion transport and metabolism (COG category P) and cell motility (COG N) were differentially expressed only in the ΔscaRNA strain and not in the Δ*cas9* strain compared to the wild-type.

Single-protein differential expression analysis identified 21 significant protein features, including Nme1Cas9 itself, which was absent as expected in the deletion mutant. Excluding Nme1Cas9, 20 proteins were differentially expressed between the Δ*cas9* mutant and the wild type, with 18 being upregulated and two being downregulated (Fig. S4A and Table S5).

Four of the 20 differentially expressed proteins, excluding Nme1Cas9 itself, were also identified as significantly differentially expressed in the previously published RNA-seq data (Heidrich et al. 2019). These four proteins include the phase-variable major outer-membrane protein PorA (NMV_0958), the phase-variable bacterial lipoprotein Blp (NMV_0031), and the ferric iron binding protein EfeO (NMV_0034) along with the peroxidase EfeB (NMV_0035). Both are encoded by a three-gene operon, which includes the permease EfeU. While the role of *efeUOB* in meningococcal iron homeostasis remains unclear, in *Bacillus subtilis*, EfeO and the permease EfeU form a minimal complex for ferric iron uptake, while EfeB, a hemoprotein, oxidizes ferrous to ferric iron and scavenges ROS to protect the cell envelope (Miethke et al. 2013). In contrast, in the ΔscaRNA proteomics dataset no significant change in EfeO abundance was detected (Fig. S4B), likely reflecting assay sensitivity and protein half-life buffering that limit detection of modest translational repression, whereas RT-qPCR and translational reporter assays capture the scaRNA-dependent effect (Figs. 5C and 8).

Other proteins involved in iron metabolism that were suppressed by Nme1Cas9 include lactoferrin-binding protein LbpB (NMV_0848) and the oxygen-independent coproporphyrinogen III oxidase HemN (NMV_0417). Upregulated proteins in the Δ*cas9* mutant further included PilG (NMV_0368), a platform protein that plays a key role in type IV pilus assembly and is required for pilus biogenesis, and PilU (NMV_0047), which modulates pilus-mediated functions (Rusniok et al. 2009, Muir et al. 2020). Likewise, compared to the wild-type the metabolic enzymes NADP-specific glutamate dehydrogenase GdhA (NMV_0661), a putative acyl-CoA thioester hydrolase (NMV_1471), 5-oxoprolinase subunit A PxpA (NMV_0256), imidazoleglycerol-phosphate dehydratase HisB (NMV_0801) and cytochrome c4 (NMV_1965) were more highly expressed in the *cas9* deletion strain. Finally, genes presumably suppressed by Nme1Cas9 included the DNA polymerase III alpha subunit DnaE (NMV_1992) and the replicative DNA helicase DnaB (NMV_1514).

The two proteins that were downregulated in Δ*cas9* were the NAD-specific glutamate dehydrogenase GdhB (NMV_0909) and the major outer-membrane protein PorA (Fig. S4A). Because PorA expression undergoes frequent slipped-strand mispairing at homopolymeric tracts, leading to stochastic phase variation independent of regulatory control, we interpret this difference as a phase-variation event rather than a reproducible Nme1Cas9-dependent effect. Gamma-glutamyl phosphate reductase ProA (NMV_1325), GdhA and DnaE were the three proteins that were significantly upregulated in the ΔscaRNA mutant compared to the wild type, indicating that they were repressed by scaRNA, while GdhB was significantly downregulated. These data thus suggest that GdhA, GdhB, and DnaE are coregulated by Nme1Cas9 and scaRNA.

### Absence of evidence for sequence-guided protein expression regulation by scaRNA

In view of the gene expression differences between the mutant and the wild-type strains, a computational assessment was conducted to test whether these differences might be attributable to previously described transcriptional or posttranscriptional mechanisms. In particular, it has previously been shown that Nme1Cas9 is capable of functioning as a scaRNA-guided RNase *in vitro*, a process which requires a minimum of 16 consecutive identical nucleotides between the scaRNA and the potential target sequence (Rousseau et al. 2018). However, computational similarity searches using IntaRNA/CopraRNA (Wright et al. 2014, Mann et al. 2017) and BLASTN (Altschul et al. 1990) failed to predict any *bona fide* interaction partners in the chromosome of strain 8013.

Likewise, it has been shown in *F. novicida* that transcriptional regulation by a scaRNA-guided PAM-dependent interaction of FnoCas9 with the 5′ UTR on the DNA template strand requires only a limited region of 9–15 bp complementarity with the scaRNA adjacent to a PAM and within 20 bp of the transcription start site (Ratner et al. 2019). Again, even with this reduced number of complementary bases required, BLASTN searches failed to identify any *bona fide* targets for scaRNA within the 5′ UTRs of differentially expressed genes in *N. meningitidis*.

Finally, in order to identify sequence motifs enriched either in the 5′ UTR, the coding region and/or the 3′ UTR of genes significantly differentially expressed in the Δ*cas9* and the wild-type strain in a hypothesis-free manner we performed MEME motif analysis in the discriminative mode (Bailey et al. 2009). Apart from the neisserial DNA uptake sequence (DUS), these analyses did not reveal any other sequence motifs that are shared by regulated genes and absent from nonregulated genes. Since the strain 8013 genome contains 1474 exact copies of the DUS and a further 682 copies with one mismatch, approximately one copy of a DUS with no more than one mismatch per coding sequence would be expected by chance (Bauriedl et al. 2020). These data therefore suggest that the mechanism by which meningococcal scaRNA, together with Nme1Cas9, affects gene expression likely differs from that previously described (Dugar et al. 2018, Rousseau et al. 2018, Ratner et al. 2019).

### Nme1Cas9 specifically binds to and cleaves meningococcal scaRNA *in vitro*

**Figure 4:**
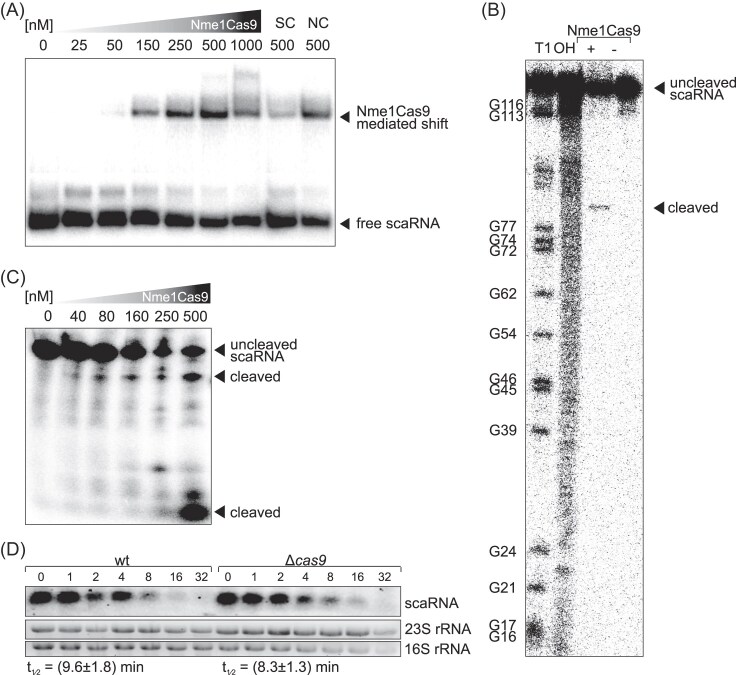
Analysis of scaRNA association with Nme1Cas9. (A) *In vitro* gel shift assay of Nme1Cas9 and scaRNA. *In vitro* transcribed and labelled scaRNA was incubated with different concentrations of purified Nme1Cas9 protein as indicated. A 200-fold molar excess of unlabelled scaRNA (specific competitor, SC) or of an unlabelled, shuffled scaRNA nucleotide sequence (nonspecific competitor, NC) was added. Arrows indicate the position of free and Nme1Cas9-bound labelled scaRNA. (B) 4 nM of labelled scaRNA was incubated in the absence (-) or presence (+) of 150 nM Nme1Cas9 protein. RNase T1 (lane T1) or partially alkali digested scaRNA (lane OH) served as nucleotide ladders. The cleavage products were analysed on a PAA/urea gel. (C) 4 nM of labelled scaRNA was incubated with different concentrations of purified Nme1Cas9 protein as indicated (0, 40, 80, 160, 250, and 500 nM) and cleavage products were analysed on a PAA/urea gel. (D) Determination of the half-life of scaRNA analysed in the wild-type and a Δ*cas9* mutant strain. The housekeeping genes 16S and 23S rRNA were used as loading controls. Further experimental details are provided in the “Methods” section. *n* = 3

**Figure 5:**
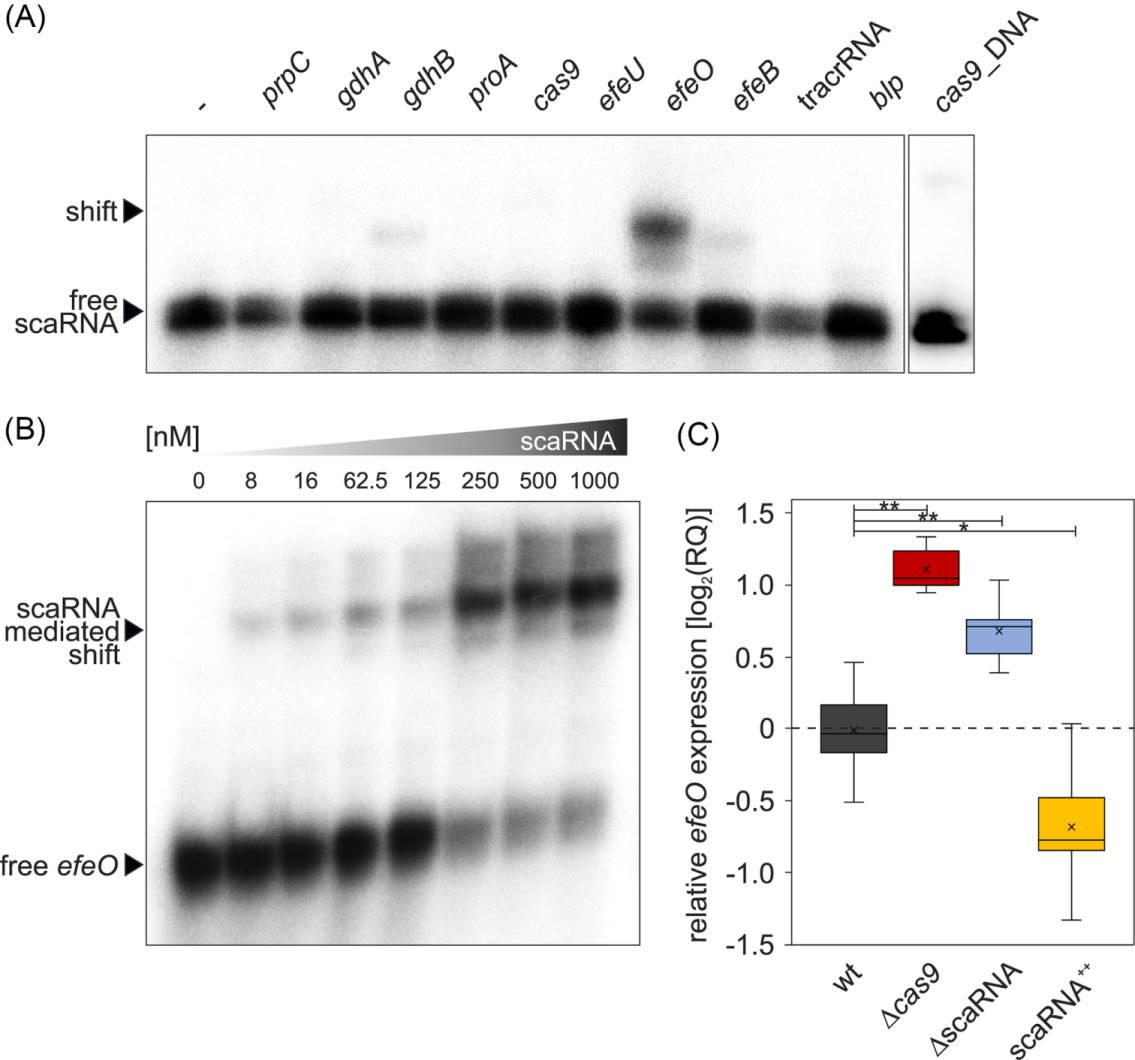
*In vitro* interaction analysis of scaRNA with potential target mRNAs and DNA using electrophoretic mobility shift assays (EMSAs). (A) *In vitro* transcribed and labelled scaRNA (4 nM) was incubated with 500 nM unlabelled mRNA/DNA targets as depicted on top of each lane. (B) *In vitro* gel shift assay of the interaction between 4 nM *in vitro* transcribed and labelled *efeO* mRNA and varying concentrations (0, 8, 16, 62.5, 125, 250, 500, and 1000 nM) of *in vitro* transcribed scaRNA. Arrows indicate the free RNAs or RNA-mediated shifts. (C) Relative quantification (RQ) of *efeO* mRNA expression change in Δ*cas9*, ΔscaRNA, and scaRNA overexpression (scaRNA^++^) strains compared to the wild-type strain using RT-qPCR. Data analysis was performed according to the ΔΔC_T_ method (Livak and Schmittgen 2001) using the housekeeping gene 16S rRNA for normalization. The data are represented as a box-whisker plot of three independent experiments. The coloured boxes represent the IQR of the data and are divided by a horizontal line representing the median. A cross marks the mean value and the whiskers outline the minimum and the maximum values of the data set. *P*-values were determined using a Wilcoxon rank-sum test comparing each mutant with the wild type. *: *P*-value < .05, **: *P*-value < .01. Data are shown for six measurements obtained from three independent experiments (*n* = 6).

In addition to crRNAs and tracrRNA, scaRNA is recovered in Nme1Cas9 co-immunoprecipitation and RNA deep-sequencing (RIP-seq) experiments (Heidrich et al. 2019), suggesting that Nme1Cas9 targets the scaRNA at the posttranscriptional level. To confirm this hypothesis, electrophoretic mobility shift assays (EMSAs) were performed, which demonstrated that scaRNA shifted in a concentration-dependent manner in the presence of Nme1Cas9 (Fig. 4A), but not tracrRNA (Fig. 5A). This indicates the formation of an Nme1Cas9–scaRNA complex *in vitro*.

To further test the specificity of the Nme1Cas9–scaRNA interaction, a 200-fold molar excess of a specific competitor RNA (SC) or a nonspecific competitor RNA (NC) was added to the binding reaction. An unlabelled scaRNA was employed as a specific competitor, whereas an unlabelled ‘shuffled scaRNA’ with a randomized nucleotide sequence, which preserved the overall nucleotide composition of scaRNA, was used as a nonspecific competitor. The addition of the nonspecific competitor did not affect the complex formation between scaRNA and Nme1Cas9 (Fig. 4A, lane NC). In contrast, the addition of an unlabelled specific competitor resulted in a reduction of the complex formation (Fig. 4A, lane SC). This finding indicates that Nme1Cas9 and scaRNA interact in a concentration-dependent and sequence-specific manner, thereby supporting previous observations from RIP-seq experiments for a physical interaction between scaRNA and Nme1Cas9 [fig. 5A in Heidrich et al. (2019)].

Recent studies have further demonstrated that Nme1Cas9 is not only capable of binding but also of cleaving single-stranded RNA *in vitro* (Rousseau et al. 2018). In line with this observation, Nme1Cas9 also exhibits concentration-dependent catalysis of scaRNA cleavage *in vitro* as illustrated in Fig. 4(B) and (C). Furthermore, a control reaction utilizing varying concentrations of Nme1Cas9 and labelled scaRNA with a shuffled nucleotide sequence demonstrated the absence of a cleavage product (Fig. S5), thereby indicating a sequence-specific cleavage of scaRNA by Nme1Cas9.

Finally, rifampicin stability assays were conducted to ascertain whether Nme1Cas9 influences scaRNA stability *in vivo*. As illustrated in Fig. 4(D), there was a modest decrease in scaRNA half-life *in vivo* in the absence of Nme1Cas9 (8.3 ± 1.3 min) compared to the wild-type (9.6 ± 1.8 min). The apparent variation in 23S and 16S rRNA band intensities likely reflects minor differences in RNA loading rather than regulation of rRNA expression, as no ribosomal proteins or associated factors were differentially expressed in our proteomic dataset (Table S5).

Together with our previous results (Heidrich et al. 2017, 2019), these data indicate that (i) Nme1Cas9 binds to scaRNA *in vitro* in a sequence-specific and concentration-dependent manner, independently of tracrRNA and PAM sequences; and (ii) Nme1Cas9 cleaves scaRNA at least *in vitro*, albeit with a nonsignificant effect on scaRNA half-life *in vivo*.

### Nme1Cas9, together with scaRNA, binds to *efeO* mRNA at the translational start site *in vitro*

Proteomic and previously published RNA-seq data for the Δ*cas9* mutant (Heidrich et al. 2019) indicated that *blp* and *efeUOB* were the most promising candidates for further study of the molecular mechanisms by which Nme1Cas9 and scaRNA repress their expression. However, given that the expression of *blp* has previously been demonstrated to be subject to phase variation because of slipped-strand mispairing (Heidrich et al. 2019) and given that the maintenance of iron homeostasis is closely linked to the oxidative stress response, we focused our analysis on the further study of the ferrous iron uptake system EfeUOB.

As the previous results suggest that Nme1Cas9 and scaRNA interact *in vitro* and probably *in vivo*, we proceeded to test their interaction also with *efeUOB* RNA *in vitro* and *in vivo*.

RT-qPCR confirmed an upregulation of *efeO* mRNA in the absence of Nme1Cas9 and scaRNA *in vivo* and showed that overexpression of scaRNA resulted in a significant downregulation of *efeO* mRNA *in vivo* (Fig. 5C). These data suggest that *efeO* is under the negative control of both Nme1Cas9 and scaRNA.

To assess whether the expression changes were due to direct interactions between Nme1Cas9 and/or scaRNA and *efeUOB*, respectively, and to identify additional target genes at the RNA level we performed EMSAs using *in vitro* transcribed 5′ UTRs along with the first 45 nucleotides of *efeU, efeO, efeB*, as well as of *cas9, blp, prpC, gdhA, gdhB*, and *proA* and ^32^P-labelled scaRNA (Frohlich and Vogel 2009, Waters and Storz 2009, Papenfort and Vogel 2010). Additionally, as previous work has described the scaRNA-guided targeting of DNA by Nme1Cas9 (Ratner et al. 2019), the complete *cas9* DNA sequence, from the promoter region to the 3′ UTR, was amplified and incubated with ^32^P-labelled scaRNA. As depicted in Fig. 5(A), only the *efeO* mRNA formed a complex with scaRNA *in vitro*, indicating a physical interaction between scaRNA and the 5′ UTR of *efeO* mRNA. Faint, nonreproducible bands occasionally appeared for *gdhB* and *efeB* in some EMSA replicates, but these lacked concentration dependence and are considered nonspecific background signals. Further EMSAs showed the formation of a concentration-dependent scaRNA–*efeO* complex (Fig. 5B), thus confirming a direct interaction between scaRNA and *efeO* mRNA. Among the tested transcripts (*efeU, efeB, cas9, blp, prpC, gdhA, gdhB*, and *proA*), only *efeO* formed a detectable complex with scaRNA under the applied conditions, indicating target specificity within this panel.

**Figure 6:**
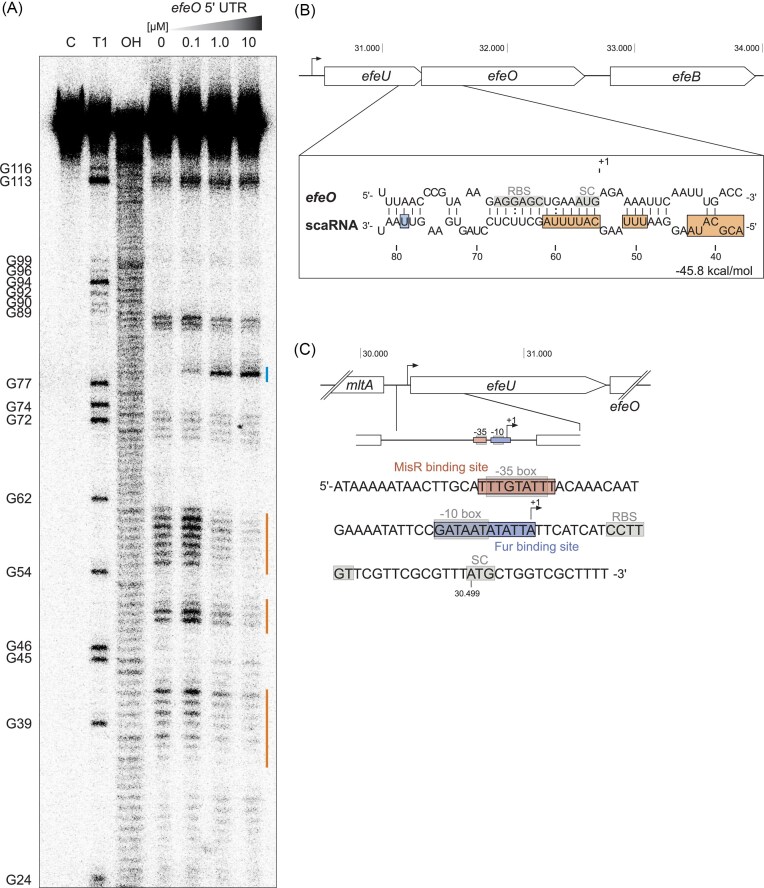
Structural and interaction site analysis of scaRNA and the 5′ UTR of *efeO* mRNA using in-line probing assay. (A) 4 nM of labelled scaRNA was incubated in the absence (0 µM) or presence (0.1, 1.0, and 10 µM) of *efeO* as indicated. Untreated scaRNA (lane C), RNase T1 (lane T1), or partially alkali digested scaRNA (lane OH) served as nucleotide ladders. Footprints mediated by the interaction between scaRNA and *efeO* are highlighted in orange. The G-numbers (G-XX) indicate the position of a guanine within the nucleotide sequence of scaRNA. (B) Genomic localization of the *efeUOB* operon and predicted interaction site between scaRNA and *efeO* using RNAhybrid (Rehmsmeier et al. 2004). The RBS and the start codon (AUG) are highlighted in gray. Experimentally validated interaction sites between scaRNA and *efeO* are highlighted in orange, while the potential scaRNA hydrolysis site is highlighted in blue. The numbers indicate the genomic position. (C) Schematic representation of the *efeU* promoter region. The RBS, the start codon (SC) and the −10 and the −35 boxes are shown in gray. Potential MisR and Fur binding sites are shown in red and blue, respectively.

**Figure 7:**
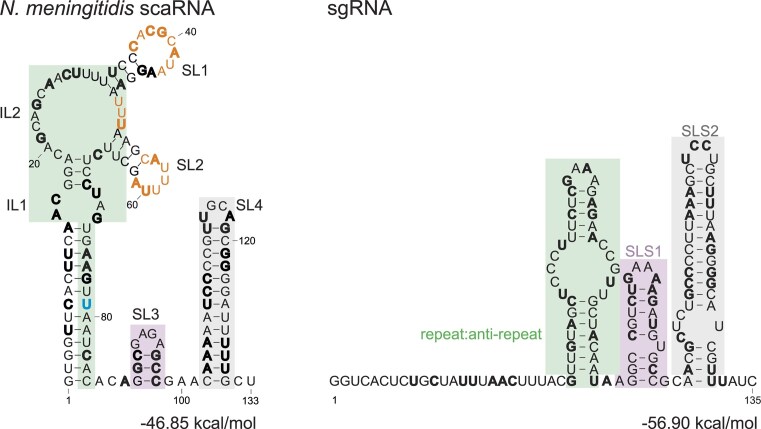
Structural analysis of sgRNA and scaRNA secondary structures. Predicted secondary structure of scaRNA using the UNAFold Web Server (Zuker 2003) and schematic representation of the sgRNA (Sun et al. 2019). For sgRNA, the repeat:antirepeat regions are highlighted in green, while the stem-loop (SL) structures (SLS) 1 and 2 are shown in pink and gray, respectively. For scaRNA, the potential repeat:antirepeat regions are highlighted in green, while the potential SLS 1 (SL 3) and SLS 2 (SL 4) are shown in pink and gray, respectively. Conserved nucleotides between sgRNA and scaRNA, identified *via* sequence alignment using MultAlin (Corpet 1988), are indicated in bold. Experimentally validated interaction sites between scaRNA and *efeO* mRNA are highlighted in orange, while the potential scaRNA hydrolysis site is highlighted in blue.

A more detailed *in silico* analysis using RNAhybrid (Rehmsmeier et al. 2004) and the full-length sequence of scaRNA together with the full-length nucleotide sequence of *efeUOB* predicted an interaction between the scaRNA and *efeO* overlapping the ribosomal binding site (RBS) and the start codon on the mRNA (Fig. 6B), suggesting a scaRNA-mediated translational inhibition of *efeO* mRNA. To test this prediction and to map the scaRNA–*efeO* interaction sites, an in-line probing assay was performed using ^32^P-labelled scaRNA and unlabelled *efeO* mRNA transcribed *in vitro* (Fig. 6A). The results of the in-line probing assay were then compared with the scaRNA secondary structure predicted by the UNAFold Web Server (Zuker 2003). As shown in Fig. 7, scaRNA folds into two internal loops (IL) and four stem loops (SL) separated by short single-stranded regions. In-line probing results showed that the addition of *efeO* mRNA resulted in a footprint in the first (SL1) and second stem loop regions (SL2) as well as in the single-stranded region separating the two stem loops (SSR2), suggesting that these regions are involved in scaRNA–*efeO* interaction, which is in line with the RNAhybrid predictions. Furthermore, the addition of *efeO* mRNA resulted in a further footprint at scaRNA nucleotide 79, suggesting that this nucleotide appears to be more susceptible to hydrolysis in the presence of *efeO* mRNA (Fig. 6A and B, marked in blue). These data suggest that the binding of the *efeO* 5′ UTR to scaRNA results in a restructuring of the scaRNA making it more susceptible to hydrolysis *in vitro*. A faint doublet signal above nucleotide U79 was consistently observed and may represent partial protection or structural stabilization of an adjacent region, although its specific role in the scaRNA–*efeO* interaction remains uncertain. Because we did not map the precise Nme1Cas9 cleavage site on scaRNA, the potential spatial relationship between the cleavage site and the scaRNA–*efeO* interaction site remains to be established.

### scaRNA reduces *efeUOB* expression *in vivo* with no detectable contribution of Nme1Cas9

To validate scaRNA-mediated translational repression of *efeO* also *in vivo* we used a translational reporter fusion system. For this purpose, the superfolder GFP (*sf-gfp*) gene (Urban and Vogel 2007, Corcoran et al. 2012) was fused to the 5′ UTR and the first 45 nucleotides of *efeO*, with the endogenous *efeO* promoter replaced by the P_tetO-1_ promoter to ensure consistent transcription initiation. This construct was integrated into the chromosome of *N. meningitidis* wild-type, Δ*cas9*, and ΔscaRNA strains at the *lctP*/*aspC* locus via homologous recombination. A PorA::GFP reporter fusion was used as a control. The meningococcal strains harbouring the EfeO::GFP and PorA::GFP reporter fusions were cultivated to mid-logarithmic growth phase in GCBL^++^ and the expression of GFP was analysed via western blot.

**Figure 8:**
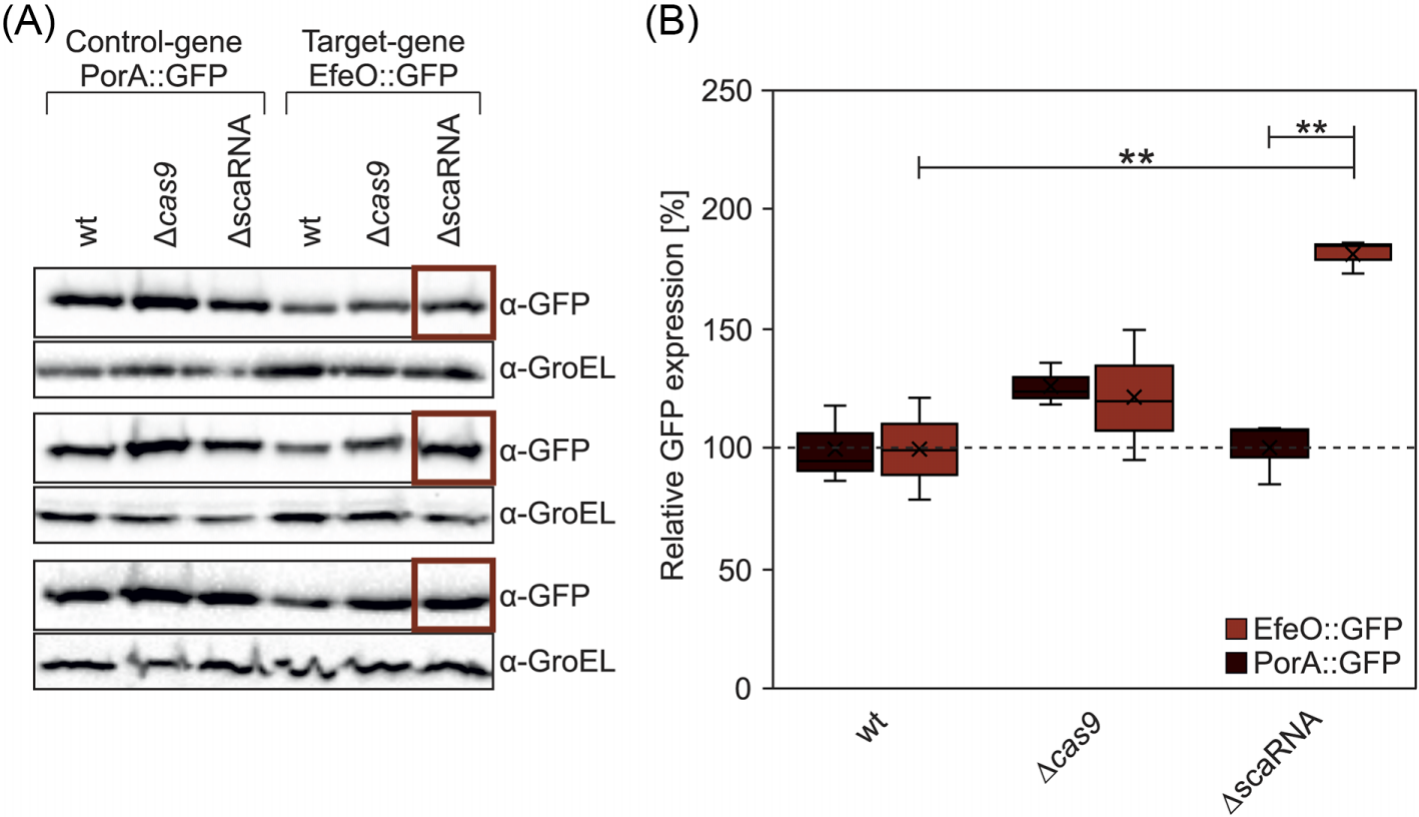
*In vivo* verification of the translational repression of *efeO* by scaRNA. (A) *N. meningitidis* 8013 wild-type (wt), Δ*cas9*, and ΔscaRNA mutant strains expressing either the control (PorA::GFP) or the EfeO::GFP fusion were grown to mid-logarithmic growth phase (OD_600nm_ = 0.5) and analysed *via* western blot. Whole-cell protein fractions were probed with anti-GFP antibody. GroEL was used as a loading control. (B) Relative change in GFP expression of the EfeO::GFP and PorA::GFP (control) fusions in the Δ*cas9* and the ΔscaRNA mutant strains, quantified from the three technical replicates shown in panel (A).

As shown in Fig. 8(A) and (B), scaRNA deletion in the presence of Nme1Cas9 resulted in approximately two-fold higher levels of EfeO::GFP expression compared to wild type, whereas *cas9* deletion had no effect on EfeO::GFP expression. These results indicate that the *efeO* 5′ UTR is sufficient for scaRNA-dependent translational repression, but not for Nme1Cas9-dependent regulation, and demonstrate that scaRNA alone can induce translational inhibition of *efeO in vivo*. Notably, PorA::GFP expression was unaffected by either *cas9* or scaRNA deletion.

In summary, these results further support the hypothesis that (i) scaRNA inhibits the translation of *efeO* in the presence of Nme1Cas9 by blocking translation initiation, and that (ii) Nme1Cas9 has no direct role in this process; it indirectly affects *efeUOB* expression by regulating the scaRNA.

## Discussion

**Figure 9:**
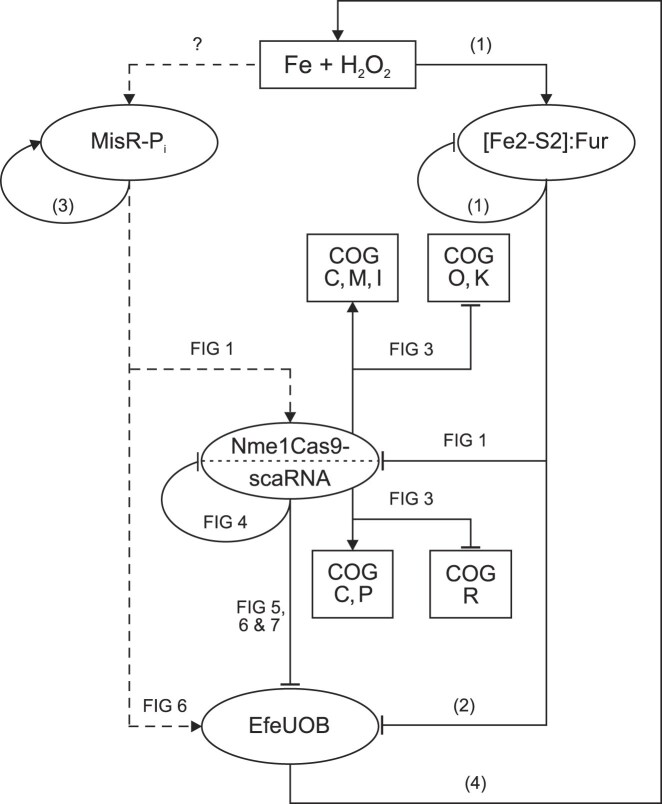
Graphical summary and working hypothesis of the Nme1Cas9–scaRNA-centred regulatory interactions in response to oxidative stress exerted via the Fenton reaction. Solid lines represent regulatory interactions for which experimental evidence is presented in the paper or the cited literature, while broken lines indicate those that have been inferred from sequence analyses. The figure numbers are provided next to each interaction line, along with the numbers referring to the respective publications as follows: (1) Troxell and Hassan (2013), (2) Basler et al. (2006) and Delany et al. (2006), (3) Tzeng et al. (2006), and (4) Cao et al. (2007). The COG functional categories that are regulated by Nme1Cas9 are given in boxes above and those regulated by scaRNA below the dotted horizontal line. COG functional categories: C, energy production and conversion; I, lipid transport and metabolism; K, transcription; M, cell wall/membrane biogenesis; P, inorganic ion transport and metabolism; Q, secondary metabolite biosynthesis, transport, and catabolism; and R, general function prediction only. Symbols: ⊥ indicates negative/inhibitory, whereas ↓ indicates positive/activating regulation. Further details can be found in the main text.

scaRNAs are a class of noncanonical small RNAs initially identified in *F. novicida*, where they regulate virulence gene expression rather than mediate adaptive immunity (Sampson et al. 2013, 2019, Guzina et al. 2018, Ratner et al. 2019, Markle et al. 2021). Given the genetic linkage between scaRNA and *cas9* as revealed by meningococcal genome comparisons (Heidrich et al. 2017), as well as a probable physical interaction between scaRNA and Nme1Cas9 as suggested by RIP-seq experiments (Heidrich et al. 2019), we hypothesized that both cooperate also functionally under infection-relevant conditions, such as iron deprivation and oxidative stress. The results of this study are summarised in Fig. 9, which also provides a hypothesis for the functional and regulatory links between the transcriptional regulators Fur and MisR, respectively, the complex of Nme1Cas9 and scaRNA, oxidative stress, iron availability, EfeO expression, and cellular response pathways.

### Potential effects of Nme1Cas9 on oxidative stress response and pilus expression

As described above and shown in Table S5, Nme1Cas9 downregulates the type IV pilus (tfp) proteins PilG and PilU, which are critical for pilus biogenesis (Pelicic 2008, Muir et al. 2020). This suggests that Nme1Cas9 (indirectly) affects pilus assembly and therefore transduction by MDAΦ (Meyer et al. 2016) as well as host cell attachment (Carbonnelle et al. 2009, Dos Santos Souza et al. 2020). Furthermore, the repression of PxpA, HemN, GdhA, HisB, and cytochrome c4 by Nme1Cas9 (Table S5) impacts oxidative stress resistance by disrupting detoxification pathways and affecting redox balance (Lushchak 2001, Seib et al. 2006). Reduced *cytochrome c4* expression impairs electron transport in the respiratory chain, while decreased *HemN* and *PxpA* expression limits heme synthesis, essential for cytochromes, catalase, and peroxidases, leading to increased ROS production. Additionally, reduced antioxidant capacity arises from diminished glutathione and histidine synthesis, due to lower expression of GdhA and HisB. The resulting disruption of redox buffering and NADPH regeneration further compromises intracellular redox balance, contributing to the reduced fitness of *cas9^+^* strains under oxidative stress and impaired host cell interactions (Fig. 2). This aligns particularly well with the observed oxidative stress resistance in Nme1Cas9-lacking strains (Fig. S3C and D) and their reduced host cell adhesion (Heidrich et al. 2019). Together with its regulatory effects on tfp biogenesis, this might affect bacterial resistance against phage infection and/or replication. However, the exact molecular mechanisms by which Nme1Cas9 exerts these regulatory effects remain elusive and additional experiments using e.g. CLIP-Seq of Nme1Cas9-bound RNAs (e.g. Heidrich et al. 2017) need to be performed under appropriate stress conditions to identify other stress-regulated ncRNAs and to assess the potential role of Nme1Cas9.

### The potential influence of Fur and MisR on *cas9* and *efeUOB* expression

The detection of Fur-binding sites in the *cas9* and *efeUOB* promoters explains their de-repression under iron limitation or hydrogen peroxide (H₂O₂) exposure (Fig. 1). Fur, a key regulator of iron homeostasis, regulates over 80 genes in *N. meningitidis*, including the *efeUOB* operon (Basler et al. 2006, Delany et al. 2006). Recent findings suggest Fur binds iron–sulfur clusters ([2Fe^2+^-2S]) and that binding of the [2Fe^2+^-2S] cluster activates its DNA-binding activity (Fontenot and Ding 2023). Oxidation of these clusters by H₂O₂ releases ferrous iron (Imlay 2006), which explains the de-repression of Fur and Fur-regulated genes like *cas9* in response to H₂O₂ exposure (Troxell and Hassan 2013). In addition, ferrous iron reacts with H_2_O_2_ in the Fenton reaction to generate hydroxyl radicals ($OH \cdot $) that can damage any biological macromolecules such as ferric iron reductases, thereby likely impairing their ability to reduce Fe^3+^ back to Fe^2+^ for Fe–S-cluster regeneration (Touati 2000). Unlike H₂O₂, intracellular superoxide (O₂⁻) generated by paraquat facilitates recycling of Fe³⁺ to Fe²⁺ (Cornelis et al. 2011, Bradley et al. 2020), preserving Fur activity and repressing *cas9* (Fig. 1).

In contrast, the activation signal of the MisR/MisS two-component system and its role in regulating *cas9* and *efeUOB* (Figs.1D and 6C), as proposed by the presence of putative MisR-binding sites in their promoter regions, remain equivocal. MisR was found to regulate the expression of iron homeostasis and oxidative stress resistance genes including the bacterioferritin operon *bfrA-bfrB* or the hemoglobin receptor *hmbR* (Grifantini et al. 2004, Newcombe et al. 2005, Tzeng et al. 2008, Zhao et al. 2010), and mutations in the MisR/MisS system resulted in heightened sensitivity to oxidative stress and envelope stressors (Tzeng et al. 2008). Since envelope stress was found to be a trigger of CRISPR RNA-mediated DNA silencing in *E. coli* (Perez-Rodriguez et al. 2011), this suggests a regulatory role of MisR/MisS in the coregulation of *efeUOB* as well as *cas9* expression and in maintaining iron homeostasis in response to e.g. phage-induced damage to the meningococcal cell envelope.

### Potential posttranscriptional regulation of scaRNA by Nme1Cas9

Regarding the regulation of scaRNA expression, we have previously shown that transcription of the scaRNA is driven by a σ^70^-like promoter and is not subject to transcriptional regulation by Nme1Cas9 or tracrRNA (Heidrich et al. 2017). Since scaRNA lacks any Fur or MisR-binding sites in its promoter regions (Fig. 1D), the finding that Nme1Cas9 binds to and modestly decreases the stability of scaRNA (Fig. 4) could explain the inverse correlation of their steady-state expression levels (Fig. 1A–C). The observation that scaRNA is sufficient to enable the ssRNA binding activity of the Nme1Cas9–scaRNA complex (Fig. 4) is furthermore consistent with previous findings that a single-stranded sgRNA is sufficient to guide Nme1Cas9 for sequence-specific binding of dsDNA (Esvelt et al. 2013). Given that Nme1Cas9 cleaves ssRNA in a crRNA-guided, tracrRNA-dependent, and PAM-independent manner (Rousseau et al. 2018), it is plausible that scaRNA may function analogously to sgRNA (Esvelt et al. 2013). This is supported by the similarity in secondary structure between scaRNA and sgRNA (Sun et al. 2019). Specifically, IL1 and IL2 of scaRNA resemble the repeat:antirepeat region of sgRNA, while stem-loops SL3 and SL4 show notable similarity to sgRNA stem-loop structures SLS1 and SLS2. Interestingly, SL1 and SL2—identified as potential interaction sites with *efeO* mRNA (Fig. 7)—lie outside the putative repeat: antirepeat region of scaRNA (Fig. 7). Notably, both the SLS1/SLS2 and the repeat:antirepeat region of sgRNA have been characterized as critical structural motifs recognized by distinct domains of the Nme1Cas9 protein (Sun et al. 2019). These findings suggest that scaRNA may possess the necessary structural and functional elements to substitute sgRNA in active Nme1Cas9 complexes, potentially enabling sequence-specific ssRNA cleavage independent of tracrRNA and PAM. Further experiments using modified scaRNA and complementary target sequences are needed to further test this hypothesis.

Our data further show that Δ*cas9* is not phenotypically equivalent to scaRNA overexpression. For example, scaRNA overexpression strongly reduces *efeO* mRNA (Fig. 5C), and the EfeO::GFP translational reporter shows scaRNA-dependent repression (Fig. 8A and B), whereas Δ*cas9* does not phenocopy these effects. Moreover, Δ*scaRNA* but not Δ*cas9* mutants display marked sensitivity to oxidative stress (Fig. 2 and Fig. S3). These discrepancies are consistent with the general properties of bacterial small RNAs, which often act through nonlinear threshold effects at translation initiation sites, such that modest changes can be buffered at the protein level and not readily detected by proteomics (Levine and Hwa 2008, Vogel and Luisi 2011, Hör et al. 2020). In addition, Cas9 has been shown in other bacteria to cleave or bind RNAs independently of adaptive immunity (Dugar et al. 2018, Strutt et al. 2018), suggesting that some scaRNA-independent contributions of Nme1Cas9 remain possible.

Together, these considerations highlight the need for future *in vivo* mapping of scaRNA processing in wild-type versus Δ*cas9* backgrounds. Future work will quantify cleaved and uncleaved scaRNA species in wild-type and Δc*as9* strains and test their differential effects on *efeO* translation. In addition, compensatory mutations within the predicted interaction region will help refine the *in vivo* interaction map and define sequence determinants of repression.

### scaRNA-specific roles in ion transport and motility

Some of the regulatory effects described above may be secondary, for example due to changes in the intracellular iron pool as outlined below. scaRNA deletion specifically affected expression of inorganic ion transport (COG P) and motility (COG N) genes, indicating distinct roles of scaRNA in iron homeostasis and bacterial motility. According to the model in Fig. 9, the entire iron transport complex *efeUOB* is transcriptionally repressed by Fur (Basler et al. 2006, Delany et al. 2006) (Fig. 6C) while only the translation of the ferric iron-binding protein EfeO is repressed by scaRNA, potentially with a modest *in vivo* contribution of Nme1Cas9. Notably, *efeUOB* is among the first Fur-regulated genes to be de-repressed in response to iron limitation, acting as a ‘fast response’ operon to fluctuating iron levels and oxidative stress (Pi and Helmann 2017). Our data suggest that scaRNA–Nme1Cas9 fine-tunes *efeUOB* expression beyond Fur regulation, specifically inhibiting EfeO translation (Fig. 8) but not the ferrous iron peroxidase EfeB (Fig. 5A). Although we did not quantify iron uptake directly, these findings are nevertheless consistent with established roles of the EfeUOB system in iron uptake and oxidative stress handling in other bacteria (Miethke et al. 2013). Because our growth assays addressed iron-replete and iron-depleted conditions, but not intermediate iron availability, our conclusions are restricted to the tested regimes and do not address whether the same phenotype extends to iron-sufficient but nonexcess conditions. The resulting increase in oxidative stress susceptibility is supported by the observation that a Δ*scaRNA* mutant grows like the wild type in H₂O₂ but fails to grow in the presence of both H₂O₂ and iron (Fig. 2D). In view of the analogous promoter architecture of *efeUOB* and *hmbR*, both are subject to coregulation and thus collaborate in the uptake and transport of heme-bound iron across the inner membrane (Perkins-Balding et al. 2004). We note that whole-cell proteomics reports steady-state protein abundance, and therefore modest or condition-specific translational repression of EfeO may not be captured under our assay conditions, whereas the translational reporter and RT-qPCR detect the scaRNA-dependent effect (Figs. 5C and 8). Consistent with this, scaRNA half-life is modestly decreased in Δ*cas9* (Fig. 4D), and no Nme1Cas9 effect is detectable on the EfeO::GFP reporter (Fig. 8A and B).

Despite multiple attempts, we were unable to obtain Δ*cas9* Δ*scaRNA* double mutants in strain 8013, suggesting either technical constraints or potential synthetic interactions. We therefore included strain MC58, which naturally lacks the entire CRISPR/Cas locus, as a CRISPR/Cas-locus-negative reference background in the oxidative-stress assay. Because MC58 is not isogenic to strain 8013, it is used for contextual comparison only and not as a genetic substitute for a constructed double mutant. In a phase-variable organism such *as N. meningitidis*, we therefore prioritise causal inference from defined isogenic genetics, isogenic complementation or overexpression, verification of engineered loci, and orthogonal mechanistic assays, while interpreting single-protein differences compatible with stochastic ON/OFF switching with caution. Our proposal that Nme1Cas9 may modulate scaRNA-dependent regulation of *efeO* should therefore be regarded as a working model supported by *in vitro* cleavage (Fig. 4B and C) and opposing transcriptional responses of *cas9* and scaRNA (Fig. 1A and C), whereas direct *in vivo* contribution of Nme1Cas9 to *efeO* translational repression was not detected in the reporter assay (Fig. 8A and B). Taken together, these findings outline a model in which scaRNA directly represses *efeO* translation, linking small-RNA control to iron homeostasis and oxidative stress adaptation in *N. meningitidis*. Extension of this analysis to additional CRISPR-positive meningococcal strain backgrounds would be valuable to determine how generalizable the observed scaRNA-dependent phenotype is beyond strain 8013.

The data further suggest some functional similarity between scaRNA and RyhB in *E. coli*. Both small regulatory RNAs have been shown to mediate posttranscriptional regulation to maintain iron homeostasis under conditions of iron limitation and oxidative stress (Masse et al. 2003). By modulating iron-homeostasis-related pathways, both RNAs may contribute to limiting the toxic effects of excess iron and oxidative stress while enabling adaptation to changing environmental conditions. However, whereas RyhB functions as an Hfq-dependent small RNA, scaRNA does not bind to Hfq (Heidrich et al. 2017) or ProQ (Bauriedl et al. 2020). Further studies are required to determine whether the observed effects of scaRNA arise from direct interactions with its regulatory targets or from secondary regulatory pathways. In particular, in silico searches using RNAhybrid suggest that additional low-affinity pairing sites may exist in other transcripts, but these potential interactions remain to be experimentally validated in future studies.

### The Nme1Cas9–scaRNA-centric gene regulatory network architecture is predicted to enhance responsiveness and robustness to oxidative stress conditions

Previous findings indicate that base-pairing noncoding RNAs (ncRNAs) are often integrated into mixed regulatory circuits responding to environmental stresses (Beisel and Storz 2010, Nitzan et al. 2017), and the regulatory mechanism imparted by ncRNAs, such as scaRNA, exhibits distinct advantages over conventional transcription factors, particularly in scenarios where prompt responses to external stimuli are imperative (Shimoni et al. 2007, Levine and Hwa 2008, Mehta et al. 2008).

The negative autoregulation of the transcriptional repressor Fur (Yu and Genco 2012) speeds the response time of Fur and Fur-regulated gene expression to changes in iron concentration, thereby increasing the robustness of the steady-state expression level with respect to fluctuations in iron concentration (Rosenfeld et al. 2002). Conjointly with Nme1Cas9–scaRNA and EfeUOB, it constitutes an incoherent type 2 feed-forward loop (I2-FFL) motif (Alon 2007). The positive autoregulation of MisR (Tzeng et al. 2006) would engender a switch-like bistable response to changes in the activation of MisR by MisS (Becskei et al. 2001, Tyson et al. 2003). In addition, the combination of Nme1Cas9–scaRNA and EfeUOB gives rise to an incoherent type 1 feed-forward loop (I1-FFL) motif (Alon 2007). As I-FFL loops are known to function as pulse generators, noise filters, and sign-sensitive accelerators (Mangan and Alon 2003), we hypothesize that the mixed Fur–Nme1Cas9–scaRNA I2-FFL accelerates EfeUOB repression in response to an increase in iron concentration, whereas the mixed MisR–Nme1Cas9–scaRNA I1-FFL accelerates EfeUOB expression in response to reduced MisS activation. Consequently, both I1-FFL and I2-FFL would provide cells with the ability to amplify signals temporarily and to quickly adapt to oxidative stress conditions and provide resilience to fluctuating input signals, allowing precise control of EfeUOB expression (Mangan and Alon 2003, Tyson and Novák 2010).

The inferences made concerning the regulatory property of the CRISPR/Cas system on EfeUOB expression in responses to changes in iron concentration imply that upon inactivation of Fur due to oxidative stress, EfeUOB expression and therefore iron import would be de-repressed quickly. The observations are consistent with the finding that the CRISPR-Cas system reduces oxidative stress resistance (Fig. 2). This, in turn, impairs evasion of the host immune response during infection while enhancing host colonization by increasing resistance to bacteriophages and promoting host-cell adhesion (Zhang et al. 2013, Heidrich et al. 2019). This trade-off could provide a rationale for the observation that the CRISPR-Cas system is present in ∼60% of *N. meningitidis* strains predominantly from carriage lineages (Joseph et al. 2011). Bacterial genes that are subject to such selective trade-offs are often located on mobile genetic elements, such as bacterial defense islands (Hussain et al. 2021), which may provide a plausible explanation for the location of the meningococcal CRISPR-Cas system on a minimal mobile element (Saunders and Snyder 2002, Joseph et al. 2011). The CRISPR-Cas system therefore functions like a mobile regulatory module operating at the core of iron homeostasis (Young 2016).

## Supplementary Material

uqag027_Supplemental_Files

## Data Availability

The mass spectrometry proteomics data have been deposited with the ProteomeXchange Consortium via the PRIDE (Perez-Riverol et al. 2022) partner repository with the dataset identifier PXD037567.
