# Supplementary material for: The CRISPR/Cas-associated scaRNA modulates efeUOB expression and stress responses in Neisseria meningitidis
Source: Microlife. 2026 Jul 20;7:uqag027. doi: 10.1093/femsml/uqag027 (PMC13431127; doi:10.1093/femsml/uqag027)
Supplement: uqag027_Supplemental_Files [file uqag027_supplemental_files.zip › Figure captions and Table titles.docx]

**Figure S1. Characterization of the small CRISPR-associated RNA (scaRNA) in *N. meningitidis* strain 8013 and in *F. novicida* strain U112.**

(A-B) Genomic organization of the type II CRISPR/Cas system in *N. meningitidis* strain 8013 (A) and in *F. novicida* strain U112 (B). The CDSs are represented by arrows and transcriptional terminators are represented by stem-loop structures. The numbers indicate the genomic position.

(C-D) Prediction of the secondary structure of scaRNA in *N. meningitidis* strain 8013 (C) and *F. novicida* strain U112 (D). The stem-loops (SL) and the internal loops (IL) are indicated. Secondary structures were predicted by the UNAFold Web Server (Zuker 2003).

Figure S2. Bacterial fitness analysis of *N. meningitidis* wild type (wt), ΔscaRNA, scaRNA complementation (scaRNA^+^) and scaRNA overexpression (scaRNA^++^) strains in EMEM^+++^.

Growth was monitored by measuring the optical density at 600 nm (OD_600nm_) as a non-destructive proxy for cell density once every hour for six hours. The time in hours is displayed on the x-axis and the OD_600nm_ on the y-axis. The data show mean values of three independent experiments and the standard error of the mean (SEM) per strain and time point were calculated. n = 3

Figure S3. Growth analysis of *Nme* 8013 wild-type, Δ*cas9*, ΔscaRNA and *Nme* MC58 wild-type strains.

The optical density (OD) was determined at 620 nm once every 30 minutes for 18 hours. The time in hours is displayed on the x-axis and the OD at 620 nm on the y-axis. The data represent mean values of five independent experiments and the standard error of the mean (SEM) is depicted for each strain and time point.

(A) Growth in response to iron limitation.

(B) Growth in rich medium without oxidative stress and iron abundancy.

(C) Growth in response to iron limitation and oxidative stress.

(D) Growth in response to oxidative stress and iron abundancy.

**Figure S4. Gene expression changes at post-transcriptional level in *N. meningitidis* 8013 mediated by *cas9* (A) and scaRNA (B).**

*N. meningitidis* wild-type, Δ*cas9* and ΔscaRNA mutant strains were cultivated to mid logarithmic growth phase (OD_600nm_ = 0.5) in EMEM^+++^. Proteins were extracted and subjected to quantitative proteomics. The results of three biological replicates were visualized in a scatter blot. The blot depicts the median log_2_-transformed label-free quantitation (LFQ) ratios on the x-axis plotted against the corresponding log_10_ LFQ intensities on the y-axis determined by mass-spectrometry (MS). Raw MS data files were analysed with the MaxQuant version 1.6.2.2. (Cox 2008). Protein features with log_2_-transformed ratios of mutant vs. control with values outside 3x IQR were considered as significantly enriched and therefore highlighted in red. For more experimental details, refer to the Methods section.

Figure S5. Control scaRNA cleavage assay mediated by Nme1Cas9.

4 nM of an *in vitro* transcribed and ^32^P-labeled scaRNA with a shuffled nucleotide sequence were incubated with varying concentration (0, 80, 160, 250 and 500 nM) of purified Nme1Cas9 protein as indicated. The cleavage products were analysed on a 10 % PAA/urea gel.

**Table S1. Bacterial strains used in this study.**

**Table S2. Plasmids used in this study.**

**Table S3. DNA oligonucleotides used in this study.**

**Table S4. Consensus p-values from the piano analysis of the proteome comparison between the wild-type and the *cas9* deletion mutants (upper part) and the scaRNA deletion mutants (lower part).**

**Table S5. Differently expressed proteins between *N. meningitidis* strain 8013 wild-type and Δ*cas9* and between wild-type and ΔscaRNA.**

Quantitative proteomics comparing the wild-type and the *cas9*, respectively, the scaRNA deletion strain. For proteome analysis, the label-free quantitation (LFQ) intensities and the significance (sig.) for each protein are given. Additionally, the NMV-gene numbers (ID) along with their gene product and their COG classification are depicted. Gene product annotations were taken from the NeMeSys database (Rusniok 2009) and COG classification scheme is based on Tatusov (2001) and Galperin (2015). The presence of an EMSA-validated *Neisseria* Fur consensus sequences within the promotors of each gene are given based on Grifantini (2003).
