# Supplementary material for: The CRISPR/Cas-associated scaRNA modulates efeUOB expression and stress responses in Neisseria meningitidis
Source: Microlife. 2026 Jul 20;7:uqag027. doi: 10.1093/femsml/uqag027 (PMC13431127; doi:10.1093/femsml/uqag027)
Supplement: uqag027_Supplemental_Files [file uqag027_supplemental_files.zip › Table S2_Supplementary Data.docx]

| **Name** | **Description** | **Resistance** | **Source** |
| --- | --- | --- | --- |
| pGCC2 | Plasmid for complementation into the *lctP*/*aspCD* genomic locus of *N. meningitidis* | Km^r^ Erm^r^ | (Zhang 2013) |
| pMR68 | Plasmid for complementation into the *trpB*/iga genomic locus of *N. meningitidis*, overexpression using anhydrotetracycline | Km^r^ Erm^r^ | (Ramsey 2012) |
| pXG-10-SF | Plasmid for cloning a translation fusion between a target mRNA 5'UTR and a *sf-gfp* | Cm^r^ | (Corcoran 2012) |
| p8013_scaRNA^+^ | Plasmid (based on pMR68) harbouring construct for generating scaRNA complementation | Km^r^ Erm^r^ | This study |
| p8013_scaRNA^++^ | Plasmid (based on pMR68) harbouring construct for generating scaRNA overexpression | Km^r^ Erm^r^ | This study |
| pXG-10_*efeO*::*gfp* | Plasmid (based on pXG-10) harbouring the translational fusion *efeO*::*sf-gfp* | Cm^r^ | This study |
| pXG-10_*porA*::*gfp* | Plasmid (based on pXG-10) harbouring the translational fusion *porA*::*sf-gfp* | Cm^r^ | This study |
| p8013_*efeO*::*gfp* | Plasmid (based on pGCC2) harbouring the translational fusion *efeO*::*sf-gfp* | Erm^r^ | This study |
| p8013_*porA*::*gfp* | Plasmid (based on pGCC2) harbouring the translational fusion *porA*::*sf-gfp* | Erm^r^ | This study |

Km^r^: kanamycin resistant; Erm^r^: erythromycin resistant; Cm^r^: chloramphenicol resistant
