# Supplementary material for: The CRISPR/Cas-associated scaRNA modulates efeUOB expression and stress responses in Neisseria meningitidis
Source: Microlife. 2026 Jul 20;7:uqag027. doi: 10.1093/femsml/uqag027 (PMC13431127; doi:10.1093/femsml/uqag027)
Supplement: uqag027_Supplemental_Files [file uqag027_supplemental_files.zip › Table S3_Supplementary Data.docx]

| **Name** | **Sequence (5' > 3')** | **Description** |
| --- | --- | --- |
| **For detection of RNAs by northern blot** | | |
| 1449 | GTGGTTCACTTCAACGGAC | *scaRNA*; for non-radioactive northern blot (NB) |
| 1450 | AGCAAAAATCCCCGCTGCAA |  |
| 1790 | TGCTGCTGTCCGTTGAAGTGAACC | *scaRNA*; for radioactive NB |
| 1791 | TCATCGGCGCTGAATCGTTTCACG | 5S *rRNA*; for radioactive NB |
| **For amplification in RT-qPCR experiments** | | |
| 236 | CATACCGTGGTAAGCGGACT | *16S rRNA* |
| 237 | TGGTCGGTACAGAGGGTAGC |  |
| 1582 | CACGGCATTCTCCCTGTTC | *cas9* |
| 1583 | CATTAGAGGCACAATTCGGCG |  |
| 1609 | AAGCTGGTGGTAACCGACA | *efeUOB* |
| 1610 | TCGCCTTTTCAATGTCGCCT |  |
| **For creating sfGFP reporter fusions** | | |
| 1768 | GTTTTATGCATCAGGCTGTCTGAAACTTTAACC | *efeO* 5'UTR cloning in pXG-10-SF, carries a *NsiI* restriction site |
| 1769 | GTTTTGCTAGCTAAGGCAAGCATCACGGACAA | *efeO* 5'UTR cloning in pXG-10-SF, carries a *NheI* restriction site |
| 1820 | GTTTTATGCATGTATCGGGTGTTTGCCCGAT | *porA* 5'UTR cloning in pXG-10-SF, carries a *NsiI* restriction site |
| 1821 | GTTTTGCTAGCAAGCGGCAGTGCGGACAATA | *porA* 5'UTR cloning in pXG-10-SF, carries a *NheI* restriction site |
| JVO-0155 | CCGTATGTAGCATCACCTTC | Verification of insert in pXG-10-SF |
| pZE-Cat/1639 | TGGGATATATCAACGGTGGT |  |
| JVO-13362/1638 | TCGCTTAATTAACGGCGGATTTGTCCTACT | Amplification of *efeO*-15th-*sfgfp* or *porA*-15th-*sfgfp* insert for cloning in pGCC2, carries a *PacI* restriction site |
| pZE-Cat/1639 | TGGGATATATCAACGGTGGT | Amplification of *efeO*-15th-*sfgfp* or *porA*-15th-*sfgfp* insert for cloning in pGCC2 |
| JVO-12665/1122 | CGAGCAATACAGCGGCAGATTTTCC | Verification of insertion in *N. meningitidis* |
| JVO-12824/1123 | CTAAACCTAAAGTGAATAGCTCACTTATCAG |  |
| JVO-12665/1122 | CGAGCAATACAGCGGCAGATTTTCC | Sequencing oligo to verify translational fusion construct in *N. meningitidis* |
| **For construction of scaRNA complementation and overexpression strains** | | |
| 1499 | GTTTTTTGATATCCGACAGATATTGTGTCACAG | Amplification of *scaRNA* complementation fragment, carries a *EcoRV* restriction site |
| 1500 | GTTTTTTGTCGACTGAACCATACATTTTGCAAG | Amplification of *scaRNA* complementation fragment, carries a *SalI* restriction site |
| 1520 | GTTTTTTGATATCGTGGTTCACTTCAACGGA | Amplification of *scaRNA* overexpression fragment; carries *EcoRV* restriction site |
| 1464 | AAAGCGCAGATGCAGGAAGC | Verification of insert in pMR68 |
| 1470 | TTAGGAGGCTTACTTGTCTGC |  |
| 1706 | ACCTTGTTGGCATAAAAAGGC | Verification of insert in *N. meningitidis* |
| 1707 | AACACAGCCATCTGCACGA |  |
| 1470 | TTAGGAGGCTTACTTGTCTGC | Sequencing oligo to verify insertion in *N. meningitidis* |
| **For creating DNA templates for T7 *in-vitro* transcription of RNAs for EMSAs and in-line probing assay** | | |
| 1566 | GTTTTTTTTAATACGACTCACTATAGGGAGG GTGGTTCACTTCAACGGAC | *scaRNA*; carries a T7 promotor sequence |
| 1567 | AGCAAAAATCCCCGCTGC | *scaRNA* |
| 1622 | GTTTTTTTTAATACGACTCACTATATACAATACGTCTAGTTTACGCCCGCGATAGAGGTTGTTCACGGAAGGGGCGAACGTACTCTTAATTACAAAATAGATAGAGAGCCCCTCTGGCTGCTTAGCAGCTACAGTTGCTTGACCACCACTATTCATAT | shuffled nucleotide sequence of *scaRNA*; carries a T7 promotor sequence |
| 1623 | ATATGAATAGTGGTGGTCAAGCAACTGTAGCTGCTAAGCAGCCAGAGGGGCTCTCTATCTATTTTGTAATTAAGAGTACGTTCGCCCCTTCCGTGAACAACCTCTATCGCGGGCGTAAACTAGACGTATTGTATATAGTGAGTCGTATTAAAAAAAAC |  |
| 1585 | GTTTTTTTTAATACGACTCACTATAGAAATGAGAACCGTTGCTACAATAAGGCCGTCTGAAAAGATGTGCCGCAACGCTCTGCCCCTTAAAGCTTCTGCTTTAAGGGGCATCGTTTA | *tracrRNA*; carries a T7 promotor sequence |
| 1586 | TAAACGATGCCCCTTAAAGCAGAAGCTTTAAGGGGCAGAGCGTTGCGGCACATCTTTTCAGACGGCCTTATTGTAGCAACGGTTCTCATTTCTATAGTGAGTCGTATTAAAAAAAAC |  |
| 1764 | GTTTTTTTTAATACGACTCACTATATTCCGATAATATATTATTCATCATCCTTGTTCGTTCGCGTTTATGCTGGTCGCTTTTTTAATTATGTTGCGCGAG | 5'UTR *efeU*; carries a T7 promotor sequence |
| 1765 | CTCGCGCAACATAATTAAAAAAGCGACCAGCATAAACGCGAACGAACAAGGATGATGAATAATATATTATCGGAATATAGTGAGTCGTATTAAAAAAAAC |  |
| 1615 | GTTTTTTTTAATACGACTCACTATATGTCTGAAACTTTAACCCGTAAAGAGGAGCTGAAATGAGAAAATTCAATTTGACCGCATTGTCCGTGATGCTTGCCTTAGGT | 5'UTR *efeO*; carries a T7 promotor sequence |
| 1616 | ACCTAAGGCAAGCATCACGGACAATGCGGTCAAATTGAATTTTCTCATTTCAGCTCCTCTTTACGGGTTAAAGTTTCAGACATATAGTGAGTCGTATTAAAAAAAAC |  |
| 1766 | GTTTTTTTTAATACGACTCACTATAACGGCGTGCAGGGATACCCATCCTGCTGCACGGATATTGAAGGAAACACCATGAGCAAAAACCAACCCGCACAACCG | 5'UTR *efeB*; carries a T7 promotor sequence |
| 1767 | CGGTTGTGCGGGTTGGTTTTTGCTCATGGTGTTTCCTTCAATATCCGTGCAGCAGGATGGGTATCCCTGCACGCCGTTATAGTGAGTCGTATTAAAAAAAAC |  |
| 1640 | GTTTTTTTTAATACGACTCACTATAACAAATCCGCATCGGTCGTCTGAAAACCCGAAACCCATAAAAACACAAAGGAGAAATACCATGACTGAAACTACTCAAACCCCGACCT | 5'UTR *prpC*; carries a T7 promotor sequence |
| 1641 | AGGTCGGGGTTTGAGTAGTTTCAGTCATGGTATTTCTCCTTTGTGTTTTTATGGGTTTCGGGTTTTCAGACGACCGATGCGGATTTGTTATAGTGAGTCGTATTAAAAAAAAC |  |
| 1778 | GGGTGTTCAGGTCAGTCATTCGGATACTCCTTTTGGTTGATAGTGTACTAATGGGAAAACAAATTTGTAGGTTATAGTGAGTCGTATTAAAAAAAAC | 5'UTR *gdhA*; carries a T7 promotor sequence |
| 1779 | GTTTTTTTTAATACGACTCACTATAACCTACAAATTTGTTTTCCCATTAGTACACTATCAACCAAAAGGAGTATCCGAATGACTGACCTGAACACCC |  |
| 1780 | GTTTTTTTTAATACGACTCACTATAGAGCGAAATGCACGCCCCTGAAAATCTTTTTGTGAAAAGGAAGCAAAATGTCTGAAGCCCTTGCAAAAGAAACCCTCAATCC | 5'UTR *gdhB*; carries a T7 promotor sequence |
| 1781 | GGATTGAGGGTTTCTTTTGCAAGGGCTTCAGACATTTTGCTTCCTTTTCACAAAAAGATTTTCAGGGGCGTGCATTTCGCTCTATAGTGAGTCGTATTAAAAAAAAC |  |
| 1774 | GTTTTTTTTAATACGACTCACTATAATTATTTGCAACGTTGTTTACGGAGTAATAAATGGCTGCCTTCAAACCTA | 5'UTR *cas9*; carries a T7 promotor sequence |
| 1775 | TAGGTTTGAAGGCAGCCATTTATTACTCCGTAAACAACGTTGCAAATAATTATAGTGAGTCGTATTAAAAAAAAC |  |
| 1717 | ATTCTAGCCGTTGTGAGATG | *cas9* DNA |
| 1741 | TTAATTTAACGGACAGGCGG |  |
| 1613 | GTTTTTTTTAATACGACTCACTATAAATATACTTACCCGGTCTTAATGTTAACGGAGTATGGAAATGAAACAAATGCTTTTAGCCGTCGGCGTGGTGGCGGTGTT | 5'UTR *blp*; carries a T7 promotor sequence |
| 1614 | AACACCGCCACCACGCCGACGGCTAAAAGCATTTGTTTCATTTCCATACTCCGTTAACATTAAGACCGGGTAAGTATATTTATAGTGAGTCGTATTAAAAAAAAC |  |
