# Supplementary material for: The CRISPR/Cas-associated scaRNA modulates efeUOB expression and stress responses in Neisseria meningitidis
Source: Microlife. 2026 Jul 20;7:uqag027. doi: 10.1093/femsml/uqag027 (PMC13431127; doi:10.1093/femsml/uqag027)
Supplement: uqag027_Supplemental_Files [file uqag027_supplemental_files.zip › Table S4_Supplementary Data.docx]

| **COG^1^** | **Distinct-directional (dn)** | **Mixed-directional (dn)** | **Non-directional (dn)** | **Mixed-directional (up)** | **Distinct-directional (up)** |
| --- | --- | --- | --- | --- | --- |
| A | 0.2561700 | 0.5908900 | 0.6509300 | NA | 0.748830 |
| C | 0.0022998 | 0.0415960 | 0.1981800 | 0.605740 | 0.997800 |
| D | 0.7427300 | 0.6401350 | 0.1891800 | 0.058844 | 0.257370 |
| E | 0.0739930 | 0.0567445 | 0.5506450 | 0.933160 | 0.926110 |
| F | 0.1221900 | 0.6396850 | 0.6510850 | 0.468050 | 0.881210 |
| G | 0.5768400 | 0.4846500 | 0.2816250 | 0.182980 | 0.417360 |
| H | 0.4166600 | 0.9588000 | 0.9608500 | 0.827020 | 0.588840 |
| I | 0.8058200 | 0.0416960 | 0.1269900 | 0.473300 | 0.194280 |
| J | 0.0834920 | 0.9819500 | 0.9998500 | 0.992000 | 0.917610 |
| K | 0.7271300 | 0.9630500 | 0.5719900 | 0.048495 | 0.280070 |
| L | 0.9065100 | 0.5287500 | 0.1973800 | 0.152680 | 0.093591 |
| M | 0.1804800 | 0.0155480 | 0.0604940 | 0.423560 | 0.803220 |
| N | 0.9286100 | 0.2106300 | 0.1924300 | 0.346065 | 0.070993 |
| O | 0.9843000 | 0.9191100 | 0.5890900 | 0.280670 | 0.016798 |
| P | 0.7110300 | 0.5516400 | 0.2946200 | 0.203730 | 0.287770 |
| Q | 0.4470600 | 0.6912300 | 0.8496650 | 0.820020 | 0.566240 |
| R | 0.7668200 | 0.2422800 | 0.2783700 | 0.489900 | 0.233280 |
| S | 0.8994100 | 0.6217400 | 0.3496650 | 0.287620 | 0.100690 |
| T | 0.5190500 | 0.6877300 | 0.8156700 | 0.773670 | 0.483750 |
| U | 0.9096100 | 0.3458650 | 0.0901910 | 0.120540 | 0.089091 |
| V | 0.3983600 | 0.4028100 | 0.5592400 | 0.670730 | 0.597840 |
| W | 0.9980000 | NA | 0.0855415 | 0.094441 | 0.054795 |
| A | 0.7249300 | NA | 0.7177800 | 0.7289800 | 0.350160 |
| C | 0.0077992 | 0.1419900 | 0.5082500 | 0.9109600 | 0.992300 |
| D | 0.4441600 | 0.6206900 | 0.7612200 | 0.7538750 | 0.556340 |
| E | 0.1472900 | 0.2170800 | 0.3263200 | 0.5624900 | 0.845420 |
| F | 0.7347300 | 0.5248500 | 0.1654800 | 0.0746425 | 0.261470 |
| G | 0.8729100 | 0.7671200 | 0.5454450 | 0.3020200 | 0.126790 |
| H | 0.3988600 | 0.8242700 | 0.7850700 | 0.5630900 | 0.607140 |
| I | 0.2518700 | 0.2305300 | 0.3432700 | 0.5889900 | 0.744030 |
| J | 0.7589200 | 0.9988500 | 0.9997000 | 0.9383100 | 0.247080 |
| K | 0.6650300 | 0.1019900 | 0.3172700 | 0.6875800 | 0.323570 |
| L | 0.4776500 | 0.2593700 | 0.1721800 | 0.2512250 | 0.514650 |
| M | 0.6349400 | 0.7371300 | 0.6818300 | 0.5031000 | 0.365160 |
| N | 0.6044400 | 0.1680300 | 0.0318970 | 0.0608940 | 0.391160 |
| O | 0.9162100 | 0.3933100 | 0.6491850 | 0.7577750 | 0.085191 |
| P | 0.1419900 | 0.0049495 | 0.0088991 | 0.2514700 | 0.856210 |
| Q | 0.3739600 | 0.1622800 | 0.3963100 | 0.6684300 | 0.617140 |
| R | 0.9669000 | 0.8975100 | 0.3591600 | 0.0393460 | 0.032797 |
| S | 0.6160400 | 0.4845000 | 0.1245400 | 0.0517450 | 0.383460 |
| T | 0.2607700 | 0.5645900 | 0.8819100 | 0.9453100 | 0.741730 |
| U | 0.1583800 | 0.2770700 | 0.5892900 | 0.8491650 | 0.847820 |
| V | 0.2218800 | 0.3509650 | 0.4235100 | 0.5790400 | 0.778220 |
| W | 1.0000000 | NA | 0.4208100 | 0.4082600 | 0.195180 |

^1^ COG classification according to Tatusov (2001) and Galperin (2015).
