# Supplementary material for: The CRISPR/Cas-associated scaRNA modulates efeUOB expression and stress responses in Neisseria meningitidis
Source: Microlife. 2026 Jul 20;7:uqag027. doi: 10.1093/femsml/uqag027 (PMC13431127; doi:10.1093/femsml/uqag027)
Supplement: uqag027_Supplemental_Files [file uqag027_supplemental_files.zip › Table S5_Supplementary Data.docx]

| **ID** | **Gene product** | **COG** | **Quantitative proteomics** | | | | ***Neisseria* Fur consensus sequence** |
| --- | --- | --- | --- | --- | --- | --- | --- |
|  |  |  | **Δ*cas9*** | | **ΔscaRNA** | |  |
|  |  |  | **LFQ** | **sig.** | **LFQ** | **sig.** |  |
| NMV_0023 | PilS2 (pilS2 cassette) | N | 2.4147 | 2 | 3.8978 | 0 | - |
| NMV_0031 | Blp (hypothetical lipoprotein) | M | 5.3352 | 2 | -3.9635 | 0 | - |
| NMV_0034 | EfeO (ferric iron binding protein) | P | 3.1291 | 2 | -0.6110 | 0 | + |
| NMV_0035 | EfeB (deferrochelatase/peroxidase) | P | 2.4649 | 2 | 0.6667 | 0 | + |
| NMV_0047 | PilU (PilT-like protein) | U N | 1.1957 | 2 | 0.6341 | 0 | - |
| NMV_0178.1 | RpmJ2 (50S ribosomal protein L36 type B) | J | 1.5323 | 2 | 0.3935 | 0 | - |
| NMV_0256 | PxpA (5-oxoprolinase subunit A) | R | 2.0261 | 2 | 0.7093 | 0 | - |
| NMV_0368 | PilG (type IV pilus biogenesis protein) | U N | 1.0760 | 2 | -0.3394 | 0 | - |
| NMV_0417 | HemN (oxygen-independent coproporphyrinogen III oxidase) | H | 2.6412 | 2 | 0.0105 | 0 | - |
| NMV_0661 | GdhA (NADP-specific glutamate dehydrogenase (NADP-GDH)) | E | 1.5259 | 2 | 1.3105 | 2 | - |
| NMV_0801 | HisB (imidazoleglycerol-phosphate dehydratase) | E | 1.3323 | 2 | 1.2332 | 1 | - |
| NMV_0848 | LbpB (lactoferrin-binding protein) | F | 1.8022 | 2 | -0.0660 | 0 | - |
| NMV_0909 | GdhB (NAD-specific glutamate dehydrogenase (NAD-GDH)) | E | -1.5972 | 2 | -2.0109 | 2 | - |
| NMV_0958 | PorA (major outer-membrane protein) | M | -6.4448 | 2 | -2.0963 | 1 | - |
| NMV_1170 | putative type I restriction-modification system M protein | V | 2.3505 | 2 | 0.5385 | 0 | - |
| NMV_1325 | ProA (gamma-glutamyl phosphate reductase) | E | 1.1550 | 0 | 1.7556 | 2 | - |
| NMV_1471 | putative acyl-CoA thioester hydrolase | I | 1.7251 | 2 | -0.4260 | 0 | - |
| NMV_1514 | DnaB (replicative DNA helicase) | L | 1.7499 | 2 | -1.5369 | 0 | - |
| NMV_1557 | RdgC (recombination associated protein) | L | 1.0909 | 2 | 1.1030 | 1 | - |
| NMV_1965 | Cyc (cytochrome c4) | C | 2.2621 | 2 | -0.2612 | 0 | - |
| NMV_1992 | DnaE (DNA polymerase III alpha subunit) | L | 1.8095 | 2 | 1.6502 | 2 | - |
| NMV_1993 | Cas9 (CRISPR/Cas system-associated endonuclease) | S | -7.8071 | 2 | 1.0647 | 1 | - |
