# Supplementary material for: The CRISPR/Cas-associated scaRNA modulates efeUOB expression and stress responses in Neisseria meningitidis
Source: Microlife. 2026 Jul 20;7:uqag027. doi: 10.1093/femsml/uqag027 (PMC13431127; doi:10.1093/femsml/uqag027)
Supplement: uqag027_Supplemental_Files [file uqag027_supplemental_files.zip › Table_S1_Supplementary_Data_new.docx]

| **Strain name** | **Relevant genotypes** | **Resistance** | **Source** |
| --- | --- | --- | --- |
| ***Neisseria meningitidis*** | All *N. meningitidis* mutant strains have 8013 backgrounds | | |
| 8013 | Wild type | - | IHM strain collection |
| MC58 | Wild type | - | IHM strain collection |
| Δ*scaRNA* | *scaRNA*::*aphA-1*, deletion of scaRNA | Km^r^ | This study |
| *scaRNA*^+^ | *scaRNA*::*aphA-1*, *trpB*::*scaRNA*::*erm*::*iga*, complementation of scaRNA | Km^r^ Erm^r^ | This study |
| *scaRNA*^++^ | *scaRNA*::*aphA-1*, *trpB*::*scaRNA*::*erm*::*iga*, overexpression of scaRNA | Km^r^ Erm^r^ | This study |
| Δ*cas9* | *cas9*::*aphA-1*, deletion of *cas9* (NMV_1993) | Km^r^ | (Zhang 2013) |
| *cas9*^+^ | *cas9*::*aphA-1*, *lctP*::*cas9*::*erm*::*aspCD*, complementation of *cas9* (NMV_1993) | Km^r^ Erm^r^ | (Zhang 2013) |
| Δ*tracrRNA* | *tracrRNA*::*aphA-1*, deletion of tracrRNA | Km^r^ | (Zhang 2013) |
| *tracrRNA*^+^ | *tracrRNA*::*aphA-1*, *lctP*::*tracrRNA*::*erm*::*aspCD*, complementation of tracrRNA | Km^r^ Erm^r^ | (Heidrich 2019) |
| *efeO*::*sf-gfp* | *lctP*::*efeO*-15th-*sfgfp*::*erm*::*aspCD*, translational fusion of *sf-gfp* and the 5’UTR and the first 15 amino acids of *efeO* (NMV_0034) in wild type background | Erm^r^ | This study |
| *efeO*::*sf-gfp* / Δ*cas9* | *lctP*::*efeO*-15th-*sfgfp*::*erm*::*aspCD*, *cas9*::*aphA-1*, fusion of *sf-gfp* and the 5’UTR and the first 15 amino acids of *efeO* (NMV_0034) in a Δ*cas9* background | Km^r^ Erm^r^ | This study |
| *efeO*::*sf-gfp* / Δ*scaRNA* | *lctP*::*efeO*-15th-*sfgfp*::*erm*::*aspCD*, *scaRNA*::*aphA-1*, fusion of *sf-gfp* and the 5’UTR and the first 15 amino acids of *efeO* (NMV_0034) in ΔscaRNA background | Km^r^ Erm^r^ | This study |
| *porA*::*sf-gfp* | *lctP*::*porA*-15th-*sfgfp*::*erm*::*aspCD*, fusion of *sf-gfp* and the 5’UTR and the first 15 amino acids of *porA* (NMV_0958) in wild type background | Erm^r^ | This study |
| *porA*::*sf-gfp* / Δ*cas9* | *lctP*::*porA*-15th-*sfgfp*::*erm*::*aspCD*, *cas9*::*aphA-1*, fusion of *sf-gfp* and the 5’UTR and the first 15 amino acids of *porA* (NMV_0958) in a Δ*cas9* background | Km^r^ Erm^r^ | This study |
| *porA*::*sf-gfp* / Δ*scaRNA* | *lctP*::*porA*-15th-*sfgfp*::*erm*::*aspCD*, *scaRNA*::*aphA-1*, fusion of *sf-gfp* and the 5’UTR and the first 15 amino acids of *porA* (NMV_0958) in ΔscaRNA background | Km^r^ Erm^r^ | This study |
| ***E. coli*** |  | | |
| DH5α | *F– φ80lacZΔM15 Δ(lacZYA-argF)U169 recA1 endA1 hsdR17(rK–, mK+) phoA supE44 λ–thi-1* gyrA96 relA1 | - | Stratagene |
| XL_1 blue | *recA1 endA1 gyrA96 thi-1 hsdR17 supE44 relA1 lac [F proAB lacIqZΔM15 Tn10 (Tetr)]* | Tet^r^ | Stratagene |

Km^r^: kanamycin resistant; Erm^r^: erythromycin resistant; Tet^r^: tetracycline resistant
